# AMIGO - Guided assignment of ^13^C-methyl labelled proteins

**DOI:** 10.1007/s10858-026-00491-4

**Published:** 2026-04-13

**Authors:** Thorben Maass, Lorena Rudolph, Thomas Peters, Alvaro Mallagaray

**Affiliations:** 1 Institute for Chemistry and Metabolomics, Centre for Structural and Cell Biology in Medicine, University of Lübeck, 23562 Lübeck, Germany; 2 Institute of Biochemistry, Centre for Structural and Cell Biology in Medicine, University of Lübeck, 23562 Lübeck, Germany

**Keywords:** Automated assignment, Selective side-chain labelling, Methyl walk, Paramagnetic NMR, Methyl-TROSY

## Abstract

Over the last 20 years, the number of large proteins accessible to protein-NMR analysis has increased significantly due to the development of selective [^1^H_,_^13^C]-methyl labelling techniques in combination with methyl-TROSY based NMR experiments. Structure-based strategies for the assignment of [¹H,¹³C]-methyl groups rely on comparing spatial constraints derived from methyl–methyl NOEs to a known three-dimensional structure of the protein of interest. Cross peaks in methyl-TROSY spectra are assigned to specific methyl groups by matching methyl–methyl NOEs, as observed for example in 4D HMQC-NOESY-HMQC spectra, with distances derived from a structural model. This process is commonly referred to as a “methyl walk”. Here, we present AMIGO (Automated Methyl assignment via Iterative Graph Optimization), a novel assignment algorithm that formalises the intuitive methyl walk procedure by constructing graphs with nodes representing specific methyl groups and edges reflecting methyl-methyl NOEs or short methyl-methyl distances in a model. “Building blocks” consisting of nodes and edges are then generated to reconcile structure-based and NOE-based graphs. Assignments are achieved through permutation and concatenation of individual “building blocks” in a modular fashion, enabling efficient computation even for large proteins. Additional experimental restraints, such as paramagnetic relaxation enhancements (PREs) or pseudocontact shifts (PCSs), can be integrated to validate and extend the assignments. The performance of AMIGO was validated using 11 proteins that had previously been assigned and 32 NOE networks that had been generated synthetically.

**Supplementary Information:**

The online version contains supplementary material available at 10.1007/s10858-026-00491-4.

## Introduction

NMR spectroscopy has become pivotal for the study of protein dynamics and interactions under near-physiological conditions. (Baldwin and Kay 2009; Barrett et al. 2013; Williamson 2013; Bax and Clore 2019) Traditionally, protein NMR has utilized ^15^N and ^13^C isotopic labelling of the protein backbone. However, assigning backbone resonances in large complexes can be challenging. The introduction of ^1^H- ^13^C methyl-transverse relaxation optimized spectroscopy (methyl TROSY) technique (Tugarinov et al. 2003) has provided a powerful alternative, enabling analyses of supramolecular complexes up to 1 MDa. (Sprangers and Kay 2007; Pederson et al. 2017; Mas et al. 2018; Shiraishi et al. 2018; Gauto et al. 2019; Mohanty et al. 2022; Krempl et al. 2023; McShan 2023) This approach requires selective [^1^H,^13^C]-labelling of amino acid methyl groups in an otherwise highly deuterated background, with efficient protocols now available in various expression systems, including *E. coli*, yeast, insect, and mammalian cells. (Gans et al. 2010; Proudfoot et al. 2019; Rossi et al. 2019; Schütz and Sprangers 2020) Early strategies for methyl group assignments employed a brute-force approach based on systematic mutagenesis (Amero et al. 2011) wherein individual methyl groups were removed and the resulting ^1^H,^13^C-HMQC spectra compared to those of the wild-type protein. Although effective, this method is both costly and time-consuming. Additionally, mutagenesis can perturb the chemical shifts of neighbouring methyl groups, complicating spectral interpretation. (Sprangers et al. 2005; Sprangers and Kay 2007). In the case that backbone assignments are available, pulse sequences transferring magnetization from backbone amides or carbonyls to side chain methyl groups can be used. (Tugarinov et al. 2003) More recently, structure-based assignment methods have become the preferred approach when high-resolution crystal structures are available. These methods correlate NOEs obtained from 3D or 4D HMQC NOESY experiments with inter-methyl distances derived from structural models. (Tugarinov et al. 2005; Wen et al. 2012; Proudfoot et al. 2016) After initial assignment of a few key methyl groups, one can “walk” from one methyl group to another in the structural model by following NOESY cross-peaks. Although this strategy is highly effective, it faces a significant challenge in large proteins with dense methyl labelling. Here, NOE networks become exceedingly complex, making unambiguous analysis difficult. In such cases, incorporating spatial restraints from multiple structural states has been proposed to resolve ambiguities arising from signal overlap or isolated methyl groups. (Mühlberg et al. 2022 a) Paramagnetic NMR (pNMR) offers an attractive avenue for increasing methyl assignment coverage. pNMR provides long-range structural restraints derived from pseudocontact shifts (PCS), paramagnetic relaxation enhancements (PRE), and residual dipolar couplings (RDC) and, therefore, complements short-range NOE data during assignment. PRE and PCS can be detected over distances of approximately in between 15 and 60 Å (Otting 2010), overcoming some of the problems associated to NOE-based methyl assignments. Several examples have demonstrated the successful integration of PCS with NOE restraints in large systems to increase the number of assignments available. (Flügge and Peters 2018; Maass et al. 2022; Mühlberg et al. 2022) Nevertheless, manually analysing complex NOE networks in conjunction with long-range restraints represents a laborious and time-intensive task.

Several algorithms for automated methyl group assignment have been developed in recent years to facilitate and accelerate the assignment process. (Pritišanac et al. 2020; Karamanos and Matthews 2023) These methods generally fall into three categories: NOE-based, pNMR-based, and hybrid approaches. For example, MAUS, (Nerli et al. 2021) MAGMA, (Pritišanac et al. 2017 a) MAGIC, (Monneau et al. 2017; Clay et al. 2022) and MethylFLYA (Pritišanac et al. 2019) algorithms rely solely on distance restraints from NOE data, while PRE-ASSIGN, (Venditti et al. 2011) Possum, (John et al. 2007) and PARAssign (Lescanne et al. 2017) make exclusive use of PCS or PRE information. It is, however, worth noting that pNMR-based algorithms have been validated on single or small protein datasets. Hybrid strategies such as those implemented in ARTINA, (Klukowski et al. 2022) AssignSLP_GUI, (Williams et al. 2022) FLAMEnGO2.0 (Chao et al. 2012, 2014) and MAP-XSII (Xu and Matthews 2013) integrate NOE and pNMR restraints. The first two software packages also leverage artificial intelligence to enhance performance. Several comparative studies demonstrated that while all methods yield satisfactory results, none achieved 100% assignment accuracy across all data sets. (Pritišanac et al. 2017 a, 2019; Nerli et al. 2021) In addition, many of the algorithms are implemented as “black boxes”. This highlights the need for users to critically assess automated assignment outcomes.

The development of AMIGO was driven by the objective of providing expert users with a method that combines high assignment accuracy and computational performance with a transparent, user-friendly, and traceable algorithm. To this end, AMIGO was designed to mimic the “methyl walk” approach commonly used by expert NMR spectroscopists when performing structure‐based methyl group assignments. Methyl walks are intuitive and inherently traceable, making it easier to identify and correct potential misassignments. Consequently, AMIGO prioritises transparency and interpretability, while still enabling de novo assignment even for large proteins.

The construction of methyl walks cannot be easily implemented within algorithms based on subgraph isomorphism matching approaches such as vf2 (Cordella et al. 2004) or McGregor, (McGregor 1982) which inherently lack the flexibility to generate such step-by-step, interpretable assignment paths. To overcome this limitation, we have implemented a novel graph matching approach that provides a traceable and user-guided decision-making framework. User-guided refers to the fact that AMIGO produces transparent, step-by-step assignment paths that can be inspected, evaluated, and refined by the user in subsequent iterations. AMIGO also introduces a new method for constructing structure-based graphs. Existing algorithms for methyl assignment typically assume that the structural model is entirely accurate, generating structure-based graphs using a uniform distance cut-off. However, such simplifications can lead to misassignments, as they neglect effects such as spin diffusion and local variations in protein dynamics that influence observed NOEs. The derived distance constraints and the corresponding structural graphs may then be inaccurate. (Orts et al. 2012) In contrast, AMIGO employs a graph building blocks (GBB)-based approach to dynamically calculate methyl-specific cut-off distances. GBB efficiently disassembles large structural and NOE graphs into smaller subunits (building blocks) for efficient comparison, reassembling them into matched graphs. This strategy explores multiple distance cut-offs per methyl group, thereby enhancing assignment accuracy.

AMIGO primarily relies on NOE networks for automatic assignment while incorporating long-range spatial restraints (e.g., PCS and PRE) to enhance performance. Assignments require a high-resolution structural model for calculations, although AMIGO can also accommodate protein ensembles. AMIGO has been successfully applied in our laboratory for the assignment of four large proteins, one monomeric and three dimeric, ranging from 62 to 72 kDa. For benchmarking, AMIGO has been validated using seven proteins from the MAGMA benchmark. Furthermore, 32 synthetically generated NOE networks have been tested. Thus, the benchmarking covers molecular weights up to 358 kDa and includes both sparsely populated NOE networks, e.g., those obtained with ILV labelling, and densely populated networks characteristic for stereoselective MIL^*proS/R*^V^*proS/R*^A and MIL^*proS*^V^*proS*^AT methyl labelling. AMIGO demonstrates exceptional performance with large proteins under dense, stereospecific labelling schemes, achieving nearly 100% assignment accuracy with high completeness. When compared with existing methods using similar inputs, AMIGO offers comparable performance, enabling the precise assignment of methyl resonances in large, multidomain proteins within just a few hours.

## Results and discussion

### AMIGO workflow

**Fig. 1:**
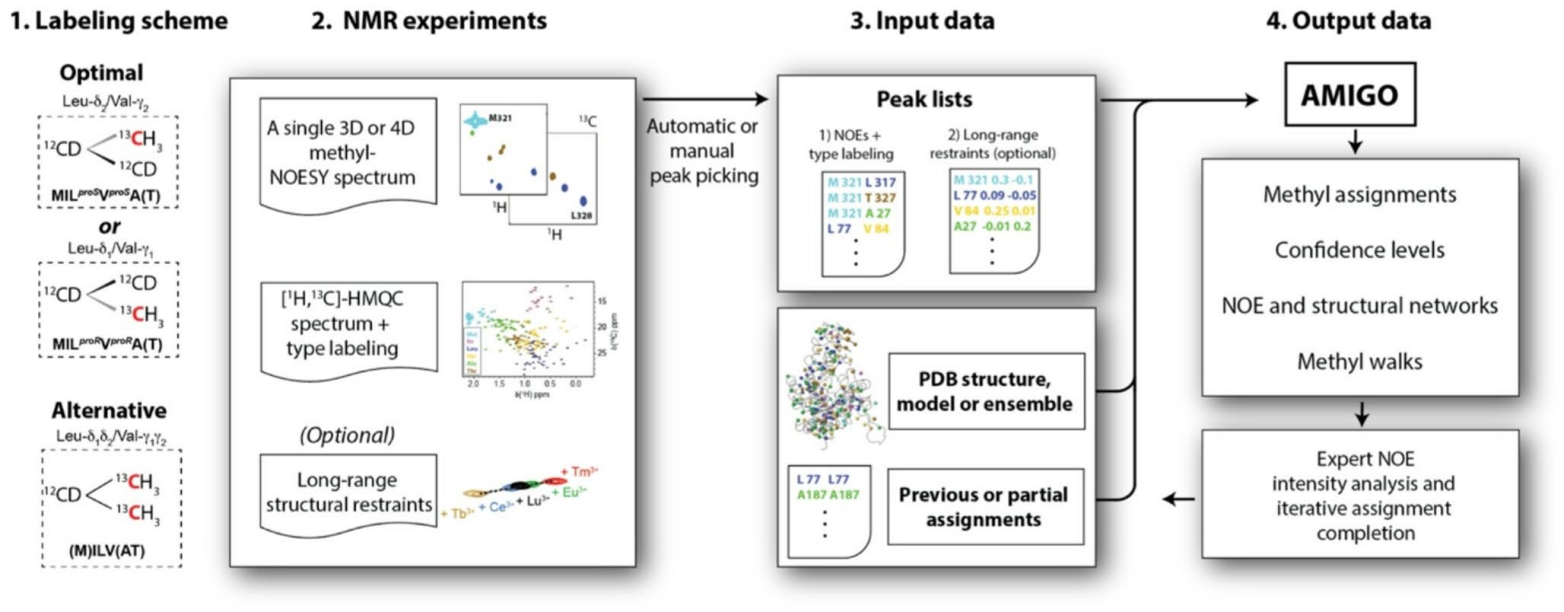
Typical workflow for automatic methyl resonance assignment with AMIGO. The basis input data required by AMIGO comprises experimental methyl-methyl NOEs from a 3D or, preferably, 4D HMQC-NOESY-HMQC experiment, information on the labelling scheme used and a high-resolution structural model. For increased performance or further refinement of the assignment, long-range structural restraints and pre-assignments can also be included

This section provides an overview of the accepted protein labelling schemes, the required NMR experiments, and the corresponding input and output data required by AMIGO. Figure 1 illustrates the process using the MIL^*proS*^V^*proS*^AT methyl labelled UDP-glucose pyrophosphorylase enzyme from Leishmania major (LmUGP) protein as example.(Mühlberg et al. 2022).

### Isotopic labelling scheme

AMIGO was developed for the backbone-independent assignment of methyl groups in large, densely and stereospecifically methyl-labelled proteins. As shown below, the best results are obtained with [^1^H,^13^C] Met-ε, Ile-δ_1_, Leu-δ_2_ and Val-γ_2_ (*proS*) or Leu-δ_1_ and Val-γ1 (*proR*) (MIL^*proS/R*^V^*proS/R*^), Ala-β (MIL^*proS/R*^V^*proS/R*^A) and Thr-γ (MIL^*proS/R*^V^*proS/R*^AT) labelling schemes. However, non-stereospecific labelling such as (M)ILV(AT) are also tolerated, where parenthesis indicate optional amino acid-type labelling.

### NMR experiments

At a minimum, AMIGO requires the acquisition of two types of NMR experiments: (i) a single 3D or 4D NMR experiment to obtain short-range spatial restraints (NOEs), and (ii) [^1^H,^13^C] HMQC spectra recorded for different labelling schemes to identify the residue type of each methyl-group signal. Additional experiments may be acquired to obtain long-range restraints, such as PCS or PRE data. For the assignment of LmUGP a single 4D HMQC-NOESY-HMQC experiment with a NOE mixing time of 180 ms was sufficient to extract short-range spatial restraints. Amino acid-type identification was achieved using [^1^H,^13^C] HMQC spectra of single amino acid-type labelled LmUGP samples. In addition, four PCS datasets were obtained from [^1^H,^13^C] HMQC spectra of MIL^*proS*^V^*proS*^AT methyl-labelled LmUGP loaded with either diamagnetic or paramagnetic lanthanide ions. Details about how NMR experiments were set-up for proteins illustrating this manuscript can be found in Table S1.

### Input data

AMIGO requires a single list of NOE cross peaks to outline a sparse NOE graph (*NG).* This list can be obtained from manual or automated inspection of the 3D or 4D NOE spectrum. The list also contains information on residue type for each peak. The algorithm explicitly considers reciprocal NOE cross-peaks for improved accuracy during graph fitting, formally analysed as directed edges in the *NG* (see Material and Methods for more information). AMIGO also accepts a second list with paramagnetic NMR (pNMR)-based long-range spatial restraints like PCS or PRE, drastically increasing the number of correct assignments and the overall algorithm performance.

A high-resolution structural model or an ensemble of structures is also required to derive a structure graph. For more details on how structural ensembles are handled by AMIGO see SI pg. 12. Additionally, a list containing pre-assignments can be provided for further refinement of the assignment. Detailed information on how to prepare the files and configure AMIGO can be found in the supplementary information.

### Output data

The primary output generated by AMIGO is a list of methyl assignments. The robustness of the assignments is evaluated by performing three full-protein replicas with different cut-off distance ranges used for the construction of the structure graphs, as will be explained later. AMIGO also provides a file containing the methyl walks together with graphical representations of NOE and structural graphs indicating the methyl walks. Methyl walk paths can be used to retrace the methyl walks proposed by AMIGO, allowing critical evaluation of the assignments (Fig. S1-S2). If long-range structural restraints are provided, AMIGO generates correlation plots of experimentally and theoretically calculated additional restraints based on the proposed assignment (Fig. S3). Deviations greater than one standard deviation usually indicate incorrect assignments due to, e.g., methyl groups in the structural model deviating from the solution conformation(s). While NOE peak intensities are not directly considered by AMIGO, they can be used by the user to further refine and disambiguate the assignment results.

Rather than treating the methyl assignments as a subgraph isomorphism problem (Pritišanac et al. 2017 a, 2019; Nerli et al. 2021), AMIGO uses a heuristic graph matching algorithm to obtain the best structural graph matching a given NMR data set. A fundamental characteristic of AMIGO is its ability to fully replicate the meticulous methyl walk strategy traditionally used by expert users. Consequently, users can backtrack through assignments with ease, enabling rapid methyl-specific evaluation. As will be explained in the next chapter, a series of weight functions allows users to improve the automated procedure iteratively, in the sense of a decision-making process.

### Automated methyl walks

The first step in a (manual) methyl walk is the assignment of one or more methyl signals to be used as starting points for assignment propagation. These so-called seminal assignments are usually obtained by comparing methyl-methyl NOE patterns to a structural model. In some cases, site directed mutagenesis may be helpful to obtain a seminal assignment. Low-abundance amino acids often give rise to unique patterns of methyl-methyl NOE cross-peaks. Therefore, in a first step the low-abundance amino acids and their theoretical NOE cross-peak patterns must be identified from the structural model. It is of advantage to use a starting amino acid methyl group that is characterized by NOEs to as many different amino acid types as possible. Once identified, one of the cross-peaks within this initial NOE cross-peak pattern is tentatively assigned and used as a next starting point. If the assignment is correct the associated NOE pattern should match the theoretical pattern obtained from the structural model. This process is repeated until no more assignments are possible.

**Fig. 2:**
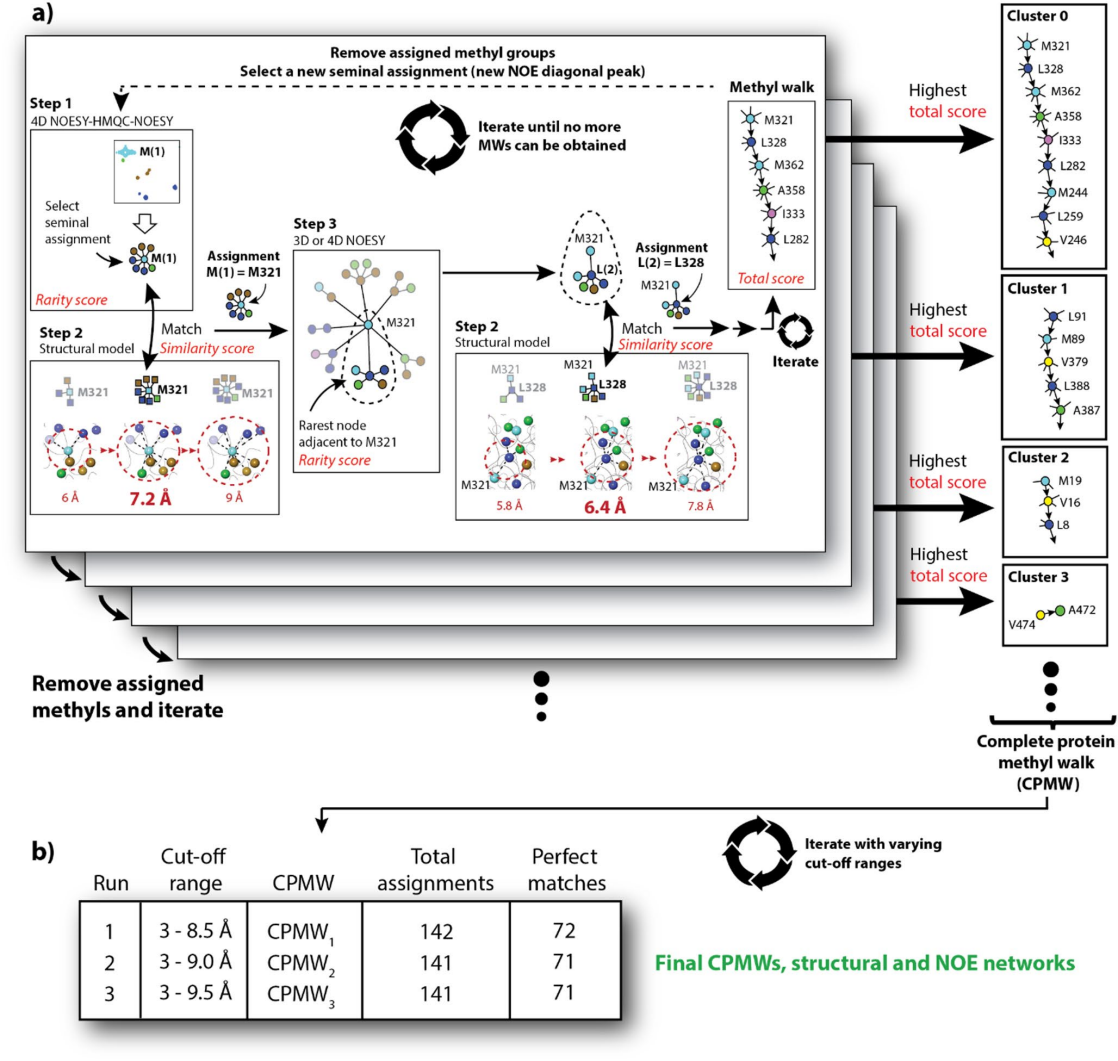
AMIGO emulates the human decision-making during a methyl-walk. Workflow of a typical protein methyl assignment performed by AMIGO

Mimicking this strategy, a typical methyl walk performed by AMIGO begins with the automated selection of a node from the NOE graph constructed from the NMR data to serve as seminal assignment. Rather than relying on a single starting point, AMIGO first ranks all methyl-group nodes according to the abundance of the respective amino acids from lowest to highest abundance, and according to the number of NOEs observed for each node from the highest to the lowest number of NOEs. Then, independent methyl walks are initiated using this ranking to select methyl groups for a seminal assignment. A node, *υ*, and all adjacent nodes are considered as a “closed neighbourhood subgraph”, denoted as *N*[*υ*]_NOE_ with the NOE subindex indicating NOESY data. AMIGO uses a *rarity score* (Eq. 1) to rank methyl groups according to the rarity of their corresponding *N*[*υ*]_NOE_ (i.e. from rarest to least rare). Therefore, the methyl group with the highest *rarity score* will be selected as seminal assignment for the first round. This is illustrated with a methionine (Met) signal M(1) in Fig. 2a, Step 1.

Then, AMIGO explores the structural model to identify the Met residue best matching the above-described requirements. Multiple closed neighbourhoods constructed from the structural model *N*[*υ*]_struct_ with variable cut-off distances within a defined range, i.e., from 3 to 9 Å in steps of 0.2 Å, are created for each Met in the structural model. Each *N*[*υ*]_struct_ is systematically compared to the experimental neighbourhood subgraph, *N*[*υ*]_NOE_, and the best match is selected. The best match corresponds to the node showing the highest *similarity score*, as defined in Eq. 4. This is exemplified in Fig. 2, Step 2, where M(1) is assigned to M321 based on the *N*[*υ*]_struct_ constructed at 7.2 Å cut-off distance. Similarly, the structural graph is initialized with the *N*[υ]_struct_ constructed at 7.2 Å around M321.

Once the seminal assignment has been performed, AMIGO advances the methyl walk by identifying the rarest NOE node adjacent to M321. The node from the NOE network showing the highest *rarity score* (Eq. 1) is then selected. In this example it is a leucine (Leu) L(2) in Fig. 2, Step 3. The corresponding *N*[*υ*]_NOE_ is extracted and systematically compared against all possible *N*[*υ*]_struct_ at variable cut-off distances around every leucine methyl group adjacent to M321. Once the best match is found (Eq. 4), the NOE node is assigned. In this example L(2) is assigned as L328. The structural graph is again expanded by comparing newly generated subgraphs *N*[*υ*]_struct_ (in this example the cut-off turned out to be 6.4 Å) to the next rarest experimental subgraph *N*[*υ*]_NOE_. This process is continued until no further assignments are possible. The procedure can be regarded as a computer-guided methyl walk, accelerating the spectral assignment.

The extent of a methyl walk and the accuracy of the assignments strongly depend on the NOE signal selected as seminal assignment. Therefore, AMIGO performs an exhaustive search by completing as many methyl walks as signals in the methyl-TROSY spectrum are available, using a new methyl group as seminal assignment in each iteration. A *total score* is calculated for each methyl walk according to Eq. 8, being the sum of all individual *similarity scores* calculated during individual methyl walks. Once every signal in the methyl-TROSY spectrum has been sampled as seminal assignment, the methyl walk with the highest *total score* is selected. This procedure allows an extensive methyl coverage, therefore ensuring the closest-to-ground solution (Fig. 2). All methyl groups corresponding to the selected methyl walk (referred to as “cluster”; see Fig. 2a) are removed from the analysis, and a new NOE cross-peak from the pool of unassigned signals is selected according to Eq. 1 as seminal assignment for a new methyl walk. Therefore, a cluster represents a subgraph of the overall NOE graph containing the methyl groups assigned together within the final methyl walk of a given iteration. The assignment is then continued with the remaining methyl groups until no further methyl walks can be obtained. This procedure generates a set of methyl walks covering only methyl groups with NOE cross peaks, namely the *complete protein methyl walk* (CPMW, see Fig. 2a). The CPMW encompasses all generated assignments, the NOE and the structural graphs. Importantly, structural graphs are constructed using methyl-specific cut-off distances that best match the NOE data.

Confidence levels for the assignments are obtained from three consecutive CPMWs with increasing cut-off ranges used for the construction of the structural graphs (Fig. 2b). Consistent assignments throughout the three iterations are classified as “safe”, while variable assignments are labelled as “ambiguous”. AMIGO will not perform assignments if a minimum “safety” criterion is not met, labelling these methyl groups as “unassigned”. A detailed explanation of these exclusion criteria is provided in the SI, p. 19. Alternatively, the metrics “number of total assignments” and “number of perfect matches” between the NOE and the structure graphs can be used to identify the best CPMW.

Key to this strategy is the use of a highly efficient algorithm able to explore large graphs with relatively low computational cost. AMIGO uses an efficient matching algorithm based on the decomposition and reassembly of complex graphs into their smallest subunits (i.e. graph building blocks or GBB) for improved performance.

### The graph building block concept

**Fig. 3:**
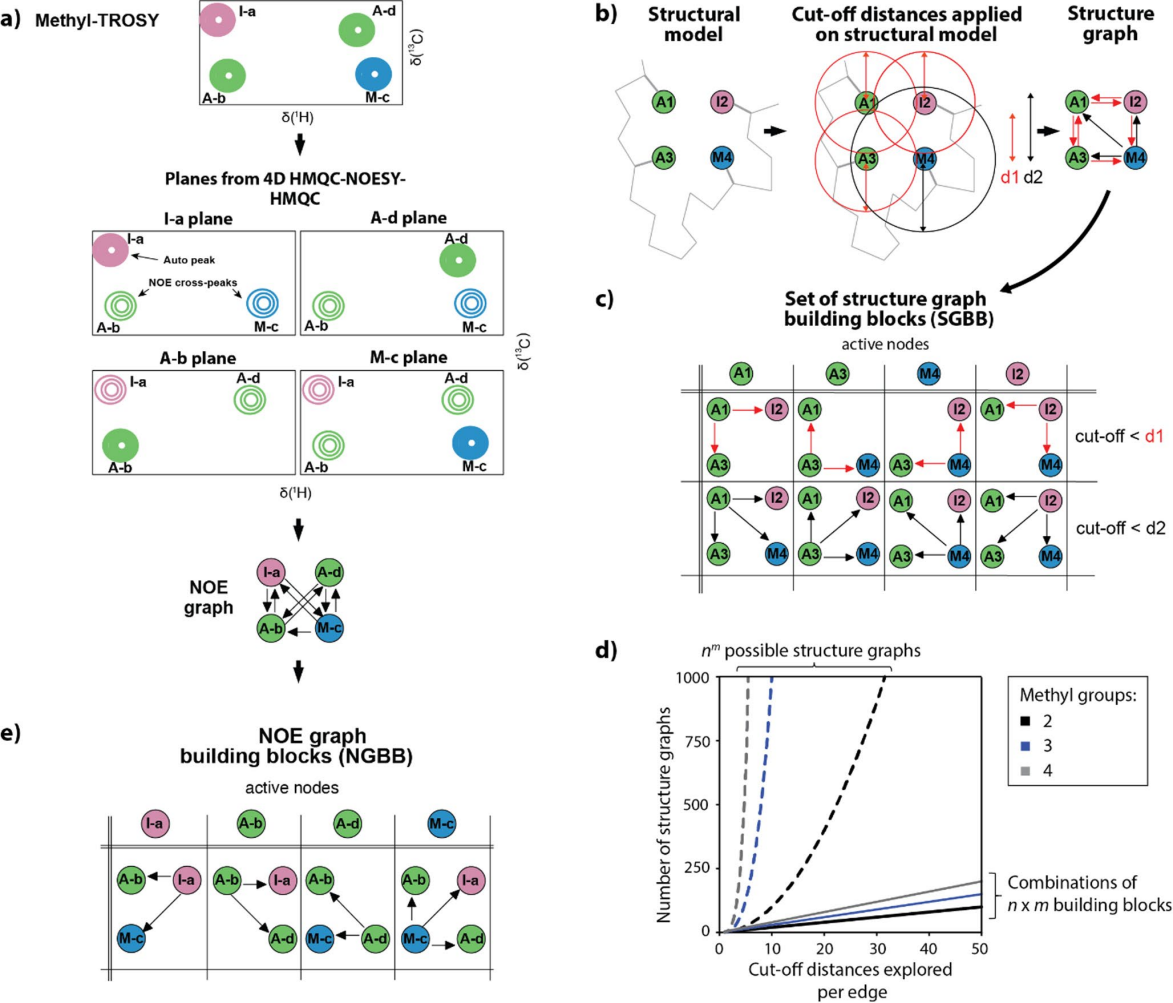
Fast graph calculation is achieved by decomposing structure and NOE graphs into GBBs. **(a)** Creation of NOE graphs. The resonances and corresponding nodes are color-coded and labelled with a capital letter according to their amino acid type and a lower-case letter as an identifier. **(b)** Creation of a structure-based graph. For each methyl group in the structural model, there is a node in the structure-based graph. Nodes are connected by edges if the methyl groups are close enough in space to allow for methyl-methyl NOEs. Factors other than the inter-nuclear distance can modulate the intensity of NOE signals. Therefore, the cut-off distances used to construct the graphs can be adjusted. Since cut-off distances are individually adjusted for each methyl group, connections between methyl groups in the graph become asymmetrical, symbolized by unidirectional edges. Methyl groups and corresponding nodes are color-coded and labelled with a capital letter according to their amino acid type and an integer as internal an identifier. It should be noted that the integer does not necessarily correspond to the amino acid position in the protein sequence. **(c)** Example for a set of structure graph-building-blocks (SGBBs). Each SGBB is characterized by an “active node” and a specific cut-off distance that is applied to the corresponding methyl group in the structural model. Note that for the example shown in (b), four methyl groups and two cut-off distances result in 2⁴ possible structure-based graphs. Assigning two arbitrary cut-off distances, d1 and d2 to every node leaves only 2 × 4 SGBBs that are required to represent the complete set of structure-based graphs. **(d)** The number of possible structural graphs as a function of the number of cut-off distances *n*. Three cases are considered for two (*m* = 2, black), three (*m* = 3, blue) and four (*m* = 4, grey) methyl groups. **(e)** The NOE graph can also be broken down into building blocks. All nodes are color-coded according to the amino acid as follows: pink for isoleucine (I), green for alanine (A), and blue for methionine (M)

In a first step, AMIGO converts the list of NOEs into a graph, yielding what will be referred to as “NOE graph” (Fig. 3a). In this graph, resonances corresponding to NOE auto-peaks (also identified in the methyl-TROSY spectrum) are depicted as nodes. Each node is characterized by a primary attribute, namely the amino acid type, which can be supplemented with additional experimental constraints such as PRE or PCS. For clarity, nodes are color-coded according to the amino acid type. The connections between nodes or the edges represent experimental NOE cross-peaks observed in multidimensional NOESY datasets. Arrows originate from the auto-peak and point towards the NOE cross-peaks. Therefore, bidirectional edges reflect a higher level of confidence than unidirectional edges.

In contrast, creating a structure graph based on a given structural model requires further considerations. Methyl groups present in the structural model are considered as nodes (see Fig. 3b). As in NOE graphs, each node is characterized by attributes, such as the amino acid type or theoretical restraints (for example, theoretical PRE or PCS). Edges between nodes depend on cut-off distances that correspond to observed NOEs. Because the magnitude of NOEs depends on many factors besides the internuclear distance, (Neuhaus 2011) specific cut-off distances may be required for each individual methyl group. By sampling various sets of cut-off distances for each methyl group, multiple structure graphs can be created and compared with the NOE graph. This variability introduces the challenge of high-dimensional data. For example, consider two possible cut-off distances, d1 and d2, for four methyl groups arranged as in Fig. 3b. Each methyl group can be correlated to either d1 or d2, resulting in 2^4^=16 possible combinations, each representing a distinct structure graph. In general, for *n* cut-off distances and *m* methyl groups, *n*^*m*^ different structure graphs can be created. As the number of methyl groups or cut-off distances increases, this approach quickly generates a vast number of structure graphs, leading to unmanageable computational times. For instance, for a protein with 100 methyl groups and assuming 5 possible cut-off distances, extrapolation of the example would result in 5^100^ (approx. 7.89 × 10^69^) possible structure graphs, a number well beyond the computational capabilities of standard computers.

Here, we introduce a simplification strategy based on graph building blocks (GBBs) to alleviate the computational challenges posed by large numbers of methyl groups or distance cut-offs (Fig. 3c). Each GBB is identified by a methyl group that serves as an “active node.” In the case of structure-based graphs, building blocks are termed SGBBs, and comprise the active node along with all methyl groups within a specific cut-off distance serving as the neighbouring nodes. As previously discussed, while *n*^*m*^ structure graphs might be created when sampling all combinations of cut-off distances, the number of possible SGBBs defining any given *N*[υ]_struct_ is reduced to *n* x *m*, hence scaling linearly with the number of methyl groups (Fig. 3d). Moreover, SGBBs can be combined to reconstruct any structure-based graph from the *n*^*m*^ set of structure graphs (see Fig. S4). This concept of GBBs naturally extends to corresponding NOE graph building blocks (NGBBs) obtained directly from NMR experiments. For example, in 4D HMQC-NOESY-HMQC experiments, each F3(^13^C)-F4(^1^H) plane contains an auto-peak that serves as the active node (Fig. 3a and e). The cross-peaks in such planes result from methyl groups that are close in space, representing the remaining nodes of a particular NGBB. Directed edges in an NGBB correspond to inter-methyl NOEs.

Like SGBBs, NGBBs can be combined to form an overall NOE graph that ideally matches only one of all possible structure-based graphs. Comparison between NGBBs and SGBBs is much more efficient than working with complete graphs, allowing an efficient construction of methyl walks. For a detailed, stepwise explanation on how methyl-walks are constructed from GBBs see SI, p. 14, Figs. S4-S11. In summary, the GBB principle encapsulates the intuitive methyl walk concept while effectively reducing the sampling space.

### Benchmarking AMIGO using stereospecifically labelled proteins

**Fig. 4:**
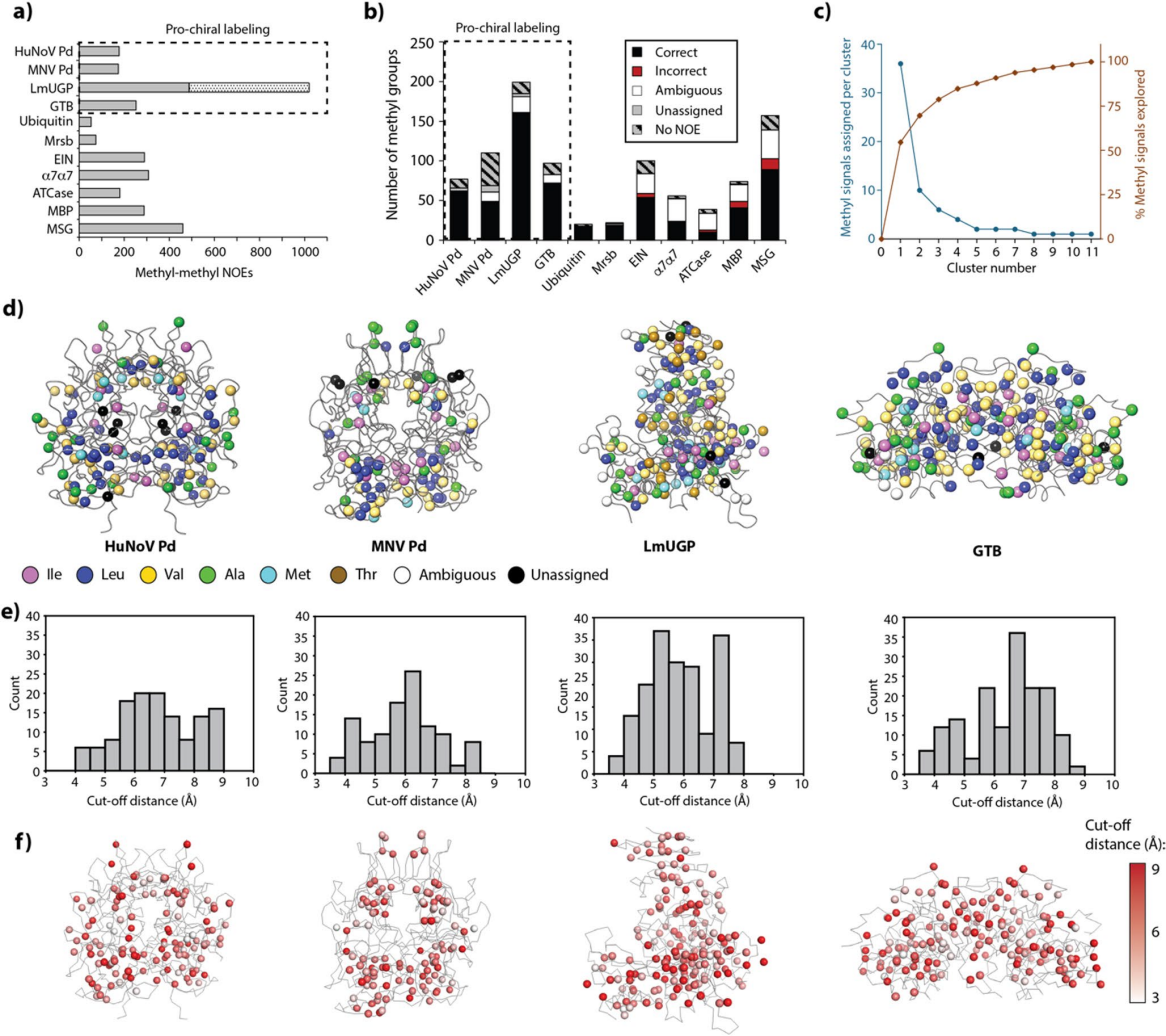
AMIGO performance. **(a)** Number of methyl-methyl NOEs available for each protein. Note that LmUGP was assigned using NOEs obtained in the open (gray) and closed conformations (dotted bar). **(b)** AMIGO performance as a function of the total number of labelled methyl groups. Proteins labelled with prochiral Leu and Val labelling (*proS*) are highlighted in a, b) with a dashed square. **(c)** Representative example showing the performance of AMIGO as a function of methyl resonances assigned per cluster (blue) and the percentage of methyl groups explored (orange) during the assignment of HuNoV Pd. **(d)** Methyl groups consistently assigned to the same amino acid in three consecutive runs by AMIGO are indicated as colored spheres, while ambiguously assigned groups are depicted in white. The colors indicate the amino-acid types of the assigned groups, with non-assigned and incorrectly assigned groups indicated in black and red, respectively. **(e)** Histograms showing methyl-specific cut-off distances selected by AMIGO for the construction of the structure-based graphs. **(f)** Representation of methyl-specific cut-off distances on crystal structures. Color-code corresponds to 3 Å (white) to 9 Å (red)

For benchmarking, AMIGO has applied to a set of 11 proteins of variable sizes, topologies and oligomeric states covering a range of stereospecific and non-stereospecific isotopic labelling schemes. The collection spans from small to very large NOE networks covering a large range of methyl densities and NOE connectivity degrees, as seen in Fig. 4a (for more details see last chapter). Importantly, AMIGO relies on NOEs for the construction of methyl walks. Therefore, although AMIGO benefits from the inclusion of long-range structural restraints, performing assignments solely on the basis of PCS or PRE data is not possible. Since we prioritise robustness over assignment extension, all methyl signals showing no NOEs were not included in the calculations. Consequently, all reported percentages refer to methyl signals exhibiting at least one NOE cross-peak, unless stated otherwise.

**Table 1:** Methyl resonance assignment statistics

	Stereospecific labelling					Non-stereospecific labelling (proteins from the MAGMA Benchmark)						
	HuNoV Pd	MNV Pd	LmUGP	GTB		Ubi	Msrb	EIN	α7α7	ATC-ase	MBP	MSG
Residues per monomer	309	302	483	297		76	142	259	233	153	370	731
Multimeric state	2	2	1	2		1	1	1	7	2	1	1
Molecular mass (kDa)	72.2	70.1	62.3	70.1		8.6	16.6	28.3	358	34.2	40.6	81.4
PDB identifier	4 × 06	6e47	2oef;4m2a	2rj1		1ubq	3e0o	1eza	1yau	1d09	1ez9	1y8b
Labelling scheme	*(proS)* MILVA	*(proS)* MILVA	*(proS)* MILVAT	*(proS)* MILVA		ILV	ILV	ILVA	ILV	ILV	ILV	ILV
NOESY dimensions	HCCH	HCCH	HCCH	HCCH		CCH	CCH	HCCH	CCH	CCH	HCCH	HCCH
Number of NOEs	175	171	485 (apo)/532 (holo)	250		53	74	290	308	181	288	460
PCS	no	yes	yes	yes		no	no	no	no	no	no	no
Protein conformations	1	1	2	1		1	1	1	1	1	1	1
All	77	100	199	97		20	22	100	56	39	73	159
With NOEs	66	59	166 (apo)/180 (holo)	83		18	21	84	53	34	71	139
Comments^A^		*+PCS*	*Apo+holo + PCS*	*+PCS*	*+loops*							
Correct	62	46	161	64	76	18	18	54	23	10	41	89
Erroneous	0	0	0	0	0	0	1	5	1	3	8	14
Ambiguous	0	10	20	0	3	0	2	25	28	21	21	36
Not assigned	4	3	4	19	4	0	0	0	1	0	1	0
Correct	57	16	164	81		17	18	64	51	24	16	33
Erroneous	4	21	11	10		3	0	17	1	5	11	9
Correct	59	24	n.c.	n.c.		16	17	56	50	18	78	97
Erroneous	0	1	n.c.	n.c.		0	0	0	0	0	0	0
Correct	-	-	-	-		-	-	0	42	8	35	0
Erroneous	-	-	-	-		-	-	0	7	4	0	0

^A^+PCS indicates NOEs and PCS, “Apo+holo + PCS” denotes that the assignments obtained in the holo form with PCS were set as constraints in the apo form, and “+loops” designates that all assignments performed with NOEs and PCS were used as constraints and AMIGO was re-run without PCS in order to assign the methyl resonances from amino acids located close to the metal centre, as discussed in the text

In a first step, AMIGO has been used for the assignment of large monomeric and dimeric proteins that had been studied in our laboratory and that have been labelled with stereospecific, highly dense MIL^proS^V^proS^A or MIL^proS^V^proS^AT labelling schemes. These proteins are the dimeric form of the protruding domain (P-dimer) of the human Norovirus capsid protein (HuNoV Pd) (Müller-Hermes et al. 2020), the corresponding P-dimer of the murine Norovirus capsid protein (MNV Pd) (Maass et al. 2022 b), the UDP-glucose pyrophosphorylase enzyme from *Leishmania major* (LmUGP) (Mühlberg et al. 2022 b), and the dimeric soluble domain from human blood group B galactosyltransferase B (GTB) (Flügge and Peters 2018) (Table 1 and S1). Assignments were validated by manual assignments and used as ground-truth for benchmarking.

The following experimental data were used for the assignment: (i) methyl-TROSY spectral peak lists generated by automated spectral peak picking in CCPNMR (Skinner et al. 2016) including amino acid type classification. (ii) NOESY peak lists generated by manual analysis of 4D HMQC-NOESY-HMQC (4D NOESY) spectra. A single 4D NOESY spectrum with optimized mixing times for maximal NOE signal count was acquired per sample. (iii) MNV Pd, GTB and LmUGP exhibit natural metal-ion binding sites permissive to lanthanoid ions. Therefore, PCSs were extracted from methyl-TROSY spectra and supplied to AMIGO as long-range spatial restraints. The corresponding set of theoretically calculated PCSs was produced using Paramagpy. (Orton et al. 2020) (iv) High-resolution crystal structures of the proteins were obtained from the Protein Data Bank with accession codes 4 × 06 for HuNoV Pd, 6E47 for MNV Pd, 2OEF and 4M2A for the open and closed conformations of LmUGP, and 2RJ1 for GTB. The *N*-, *C*-termini and the so-called internal loop from GTB as well as the *C*-terminus from LmUGP lacked electron density in the crystal structures and were modelled using ModLoop (Fiser and Sali 2003), as previously described in their corresponding publications.

When applied to the viral protein HuNoV Pd (~ 72 kDa), AMIGO correctly assigned 94% of methyl resonances, with no erroneous or ambiguous assignments (Fig. 4b; Table 1). A closer inspection of AMIGO’s assignment strategy reveals that most assignments are concentrated in the top-ranking graph matches (Fig. 4c). Specifically, an analysis of the number of assignments per cluster shows that over 75% of all assignments originate from the first four methyl walk clusters. This pattern is consistently observed across the benchmark set, indicating that focusing on the top-ranking clusters is sufficient to recover most correct assignments, therefore substantially reducing computational time, particularly for large systems.

**Fig. 5:**
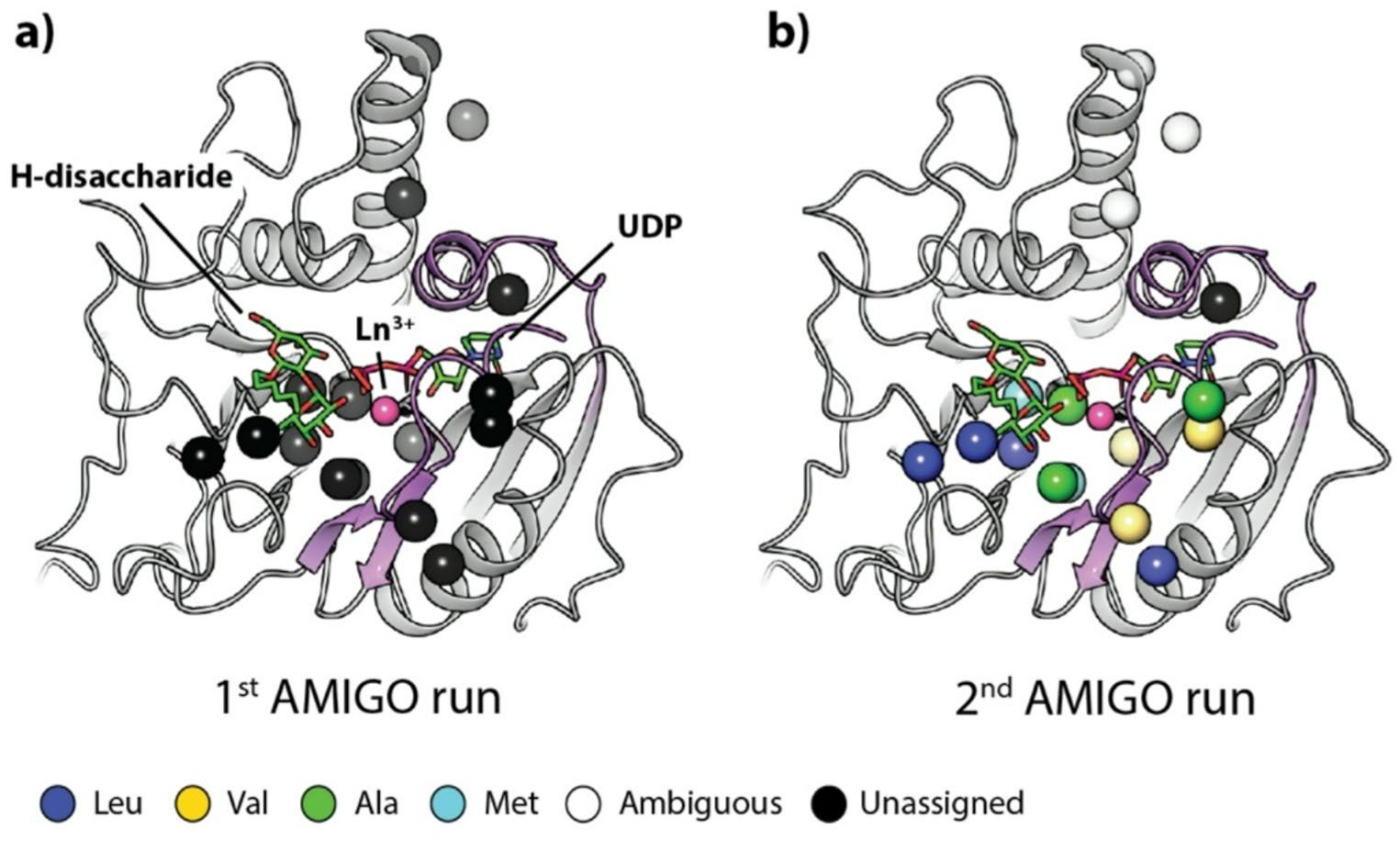
Detail of assignment of methyl groups in the GTB glycan binding pocket. **(a)** Sixteen methyl groups near the paramagnetic metal were not assigned in the initial AMIGO run. **(b)** Setting assigned methyl groups as pre-assignments and re-running AMIGO without PCS allowed to assign 12 out of 16 additional methyl groups. The crystal structure corresponds to 2RJ1, with the *C*-terminus and the internal loop modelled with ModLoop shown in violet

In the case of MNV Pd (~ 70 kDa), AMIGO assigned 69% of methyl resonances based solely on NOE cross-peaks. MNV Pd contains one metal-binding site per monomer with high affinity for lanthanoids, enabling PCS extraction. Inclusion of PCS extended the assignment to 78% (Fig. 4b; Table 1). LmUGP (monomeric, ~ 62 kDa) represents an interesting case study, as full protein assignment was only achieved through the combination of two 4D NOESY datasets acquired from different protein conformations induced by uridine diphosphate glucose (UDP-Glc) binding (Mühlberg et al. 2022 b). Using either the apo (open) or the UDP-Glc bound (closed) conformation alone enabled assignment of 38% and 66% of methyl signals, respectively. The larger number of assignments in the closed form is explained by the adoption of a more compact structure that facilitates the observation of NOE signals. PCSs could only be obtained from the closed conformation, as lanthanoids coordinate the oxygen atoms of the pyrophosphate group in UDP-Glc. Inclusion of PCS expanded the number of assignments to 81%. Using these assignments as restraints and running again AMIGO on the apo form expanded the number of correct assignments to 86% (Fig. 4b; Table 1).

Finally, we challenged AMIGO with the homodimeric enzyme GTB (~ 70 kDa). The use of NOEs alone yielded 34% assignments, indicating the necessity of additional long-range structural information. GTB requires Mn²⁺ as a cofactor, which can be substituted against lanthanoids to induce PCS. Inclusion of three PCS datasets with varying tensor orientations and magnitudes increased the assignment to 77%. Notably, 16 methyl resonances near the metal-binding site remained unassigned due to internal safeguards in AMIGO that prevent assignments when measured and predicted PCS strongly deviate (Fig. 5a). While useful in previous cases, these safeguards were detrimental here, likely due to a local conformational deviation between the structures observed in solution and in the crystal structure. To resolve this, all previously assigned methyl resonances were fixed as restraints, and AMIGO was re-run without PCS data. This enabled the assignment of 12 out of the 16 previously unassigned methyl signals, increasing the total count to 90% (Figs. 4b and 5b; Table 1). Detailed information about these safeguards and troubleshooting can be found in SI, p. 19.

### Applying AMIGO to assign non-stereospecifically labelled proteins

It is important to emphasize that AMIGO was not originally designed to handle non-stereospecifically labelled datasets. In particular, AMIGO cannot differentiate between *proS* and *proR* methyl resonances of Leu and Val residues. Consequently, when crystal structures lack the resolution to distinguish between prochiral methyl groups, AMIGO may generate ambiguous or even incorrect assignments. To evaluate this limitation, we challenged AMIGO with a set of seven proteins labelled using non-stereospecific, low-density ILV or ILVA labelling schemes. More details on this dataset originally published with MAGMA (Pritišanac et al. 2017 b) can be found in Table 1 and in Table S1.

Despite this limitation, AMIGO achieved assignment rates in methyl groups showing NOEs of 100% for Ubiquitin (~ 9 kDa), 86% for Msrb (~ 17 kDa), 77% for EIN (~ 28 kDa), 55% for α7α7 (~ 358 kDa), 65% for ATCase (~ 34 kDa), 67% for MBP (~ 41 kDa), and 63% for MSG (~ 81 kDa) (Table 1). Notably, AMIGO preferentially returned “ambiguous” rather than incorrect assignments when chirality could not be resolved from the available data. Detailed analysis revealed that 83% of erroneous assignments involved Leu or Val methyl groups, with the remaining 17% corresponding to isolated Ile residues exhibiting few NOEs to Leu or Val methyls. These results indicate that while AMIGO is not optimal for non-stereospecifically labelled systems, it still offers value as a complementary tool to guide manual curation or validation of assignments obtained via alternative algorithms.

To summarize, AMIGO demonstrates excellent performance in the assignment of stereospecifically labelled methyl groups in large proteins, particularly when combined with complementary long-range structural information such as PCS. This integrative approach is especially valuable for challenging systems where standard approaches may fail. No erroneous assignments were made across any of the stereospecifically labelled proteins when all input parameters were combined, underscoring the robustness of the algorithm. Our results also emphasize the importance of AMIGO’s adaptive algorithm, which allows the use of variable cut-off distances during structure graph construction. This is reflected in Fig. 4e, f, which displays the histogram and topological distribution of methyl-specific cut-off distances selected automatically by AMIGO for optimal graph matching. Clearly, relying on a fixed, global cut-off distance is a simplification that may lead to systematic assignment errors.

### Comparison with previous methods - Benchmarking

AMIGO was benchmarked against MAP-XSII, MAGMA, and FLAMEnGO2.0 (Table 1). Comparison with MAUS was not possible, as it requires two NOESY datasets with short and long mixing times to distinguish *proS* and *proR* methyl resonances of Leu and Val residues, which were unavailable for our examples. Similarly, MethylFLYA relies on dual NOESY datasets and performs peak picking directly from the spectra, substantially increasing the number of NOESY cross-peaks used for graph construction and thereby preventing a fair comparison focused solely on graph-matching performance. MAGIC was also not included, as it requires NOE signal intensities, which were not available in our datasets.

MAP-XSII utilizes Metropolis Monte Carlo sampling to optimize scoring functions, accepts additional long-range structural constraints (e.g., PCS and PRE), and integrates chemical shift predictions to enhance assignment coverage, albeit at an increased risk of erroneous assignments. In our benchmarking, AMIGO consistently outperformed MAP-XSII on ten out of eleven proteins tested, achieving higher numbers of correct assignments with fewer errors. A similar performance advantage was observed compared to FLAMEnGO2.0. The only exception was α₇α₇, where AMIGO conservatively classified many assignments as ambiguous due to limitations arising from non-stereospecifically labelled data.

The comparison between AMIGO and MAGMA is particularly insightful, as both algorithms depend solely on single NOESY dataset without requiring NOE intensities or chemical shift predictions. For stereospecifically labelled proteins, MAGMA provided comparable results to AMIGO for HuNoV Pd, but it assigned only half the methyl resonances for MNV Pd. Moreover, MAGMA failed to produce results for GTB and LmUGP even after 48 h of runtime on an Intel^®^ Core™ i9-9900 K Processor (3.6 GHz). Regarding non-stereospecifically labelled datasets, AMIGO yielded more correct assignments than MAGMA, although accompanied by some misassignments. Importantly, AMIGO mitigates this trade-off by explicitly reporting ambiguous assignments and providing transparent methyl walk traces for validation purposes.

Taken together, these results establish a solid foundation for exploring how AMIGO’s efficiency and accuracy are influenced by varying the number of methyl groups and labelling schemes, as discussed in the following section.

### Performance of AMIGO using experimental and simulated NOE networks from PDB structures

**Fig. 6:**
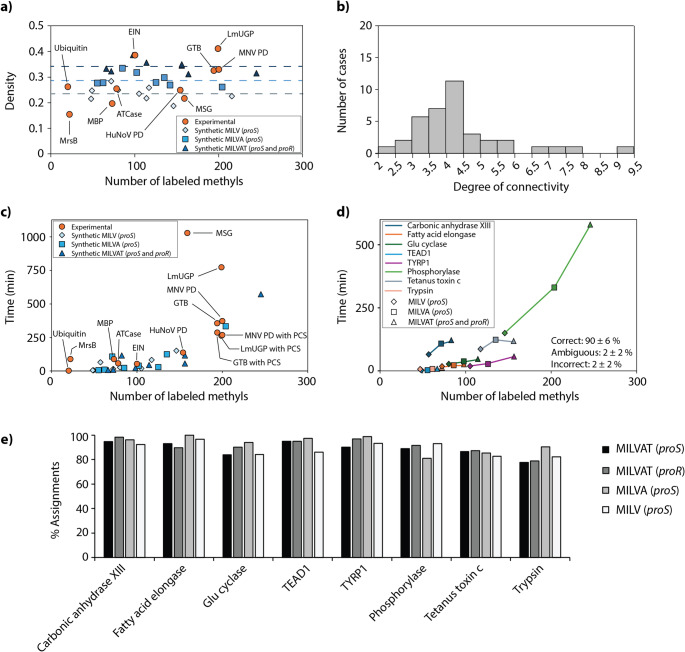
Performance of AMIGO in experimental and synthetic NOE networks as a function of labelling density. **(a)** Density distribution (defined as number of amino acids/methyl groups) of 10 experimental (orange) and 32 synthetic (blue) NOE graphs used to test AMIGO performance. Labelling schemes explored in the synthetic data are: MIL^*proS*^V^*proS*^ (pale blue diamonds), MIL^*proS*^V^*proS*^A (blue squares), MIL^*proS*^V^*proS*^AT and MIL^*proR*^V^*proR*^AT (dark blue triangles). Dashed lines indicate average density distributions for the corresponding labelling schemes. **(b)** Bar plot showing the distribution of degree connectivity (defined as 2 x number of edges/number of nodes) across experimental and synthetic NOE graphs. **(c)** Scatter plot displaying the computation time (in minutes) required by AMIGO to perform three exhaustive enumerations of possible methyl assignments, including the optimization pre-runs. The assignment of synthetic MIL^*proR/S*^V^*proR/S*^AT NOE networks from α7α7 required 33 h and are not included in this comparison. **(d)** Detailed breakdown of run times for synthetic NOE graphs as a function of the methyl labelling scheme. **(e)** Percentage of correct assignments obtained for methyl groups showing NOEs in synthetic networks as a function of the labelling scheme

The influence of protein morphology and isotopic labelling schemes on the performance of AMIGO was investigated through extensive simulations. Eight proteins with molecular weights ranging from approximately 28 kDa to 118 kDa were examined, each under four distinct isotopic labelling schemes: MIL^*proS*^V^*proS*^, MIL^*proS*^V^*proS*^A, MIL^*proS*^V^*proS*^AT and MIL^*proR*^V^*proR*^AT, resulting in a total of 32 simulated NOE networks (Table S2). Simulated data graphs were constructed by systematic random removal of edges from the corresponding structure graphs, emulating realistic experimental conditions and resulting in datasets with densities between 1.75 and 2.1 observed NOEs per methyl resonance, as previously described (Nerli et al. 2021) (Fig. 6a, b).

The results demonstrated that AMIGO efficiently manages the computational complexity inherent in graph matching under these realistic conditions, providing accurate and reliable methyl assignments within reasonable computation times, even for large proteins employing dense labelling schemes. Although increased labelling density moderately increased computational demands, AMIGO completed most assignments within 5 h using a single AMD EPYC 7302P 16-core processor with 1497.962 MHz CPU. Specifically, simulations consistently yielded high assignment rates (82–97%) across all tested isotopic labelling schemes, including proteins containing up to 245 methyl groups (Fig. 6c-e). Additionally, complete assignments for ten proteins using experimental data were accomplished in less than 24 h, further highlighting the computational practicality of the AMIGO approach.

## Conclusions

AMIGO employs a novel approach based on the use of graph building blocks to construct structure- and NOE-based graphs, enabling the assignment of proteins up to ~ 118 kDa with reasonable computational effort. The method represents an automated implementation of the classic methyl walk strategy. The accuracy of AMIGO is influenced by the type and density of the methyl-labelling scheme selected, the size of the cut-off steps selected for the construction of the structure graph building blocks, and the nature of the protein under investigation. The inclusion of long-range structural restraints like PCS or potentially also PRE significantly increases accuracy. The strength of the approach lies in the ability of AMIGO to propose potential methyl walks, which can be easily explored by the user. The performance of AMIGO has been demonstrated for the assignment of 11 proteins and 32 simulated NOE networks from PDB structures.

Future improvements could incorporate advanced tree-based optimization methods—such as Monte Carlo Tree Search to balance exploration and exploitation of methyl walk paths and Dynamic Beam Search with adaptive beam-width control—to enhance computational efficiency and mitigate being trapped in local optima. Additionally, hybrid graph neural network–guided search heuristics and Bayesian-optimized, topologically differentiated cut-off selection offer promising strategies to further improve assignment accuracy and scalability without altering the core methyl walk framework.

## Materials and methods

### Scoring functions used by AMIGO

#### The rarity score

The *rarity score* indicates the rarity of a given NGBB’s active node within the graph being considered. A higher score is obtained for infrequent amino acid types with connections to other infrequent amino acid types within that NGBB, as compared to the rest of the graph.

Consequently, the higher a given NGBB’s *rarity score*, the ‘rarer’ it is. The *rarity score* is defined in Eq. 1 as:

1 $$\eqalign{ \:rarity\:score&(NGBB,{G_{NMR}}) = \cr & - p_{active}^4\prod \: _{i = 1}^k{p_{neighbor,i}} \cr}$$

where *k* is the count of nodes neighbouring the active node within an NGBB, which is part of a collection referred to as $\:{G}_{NMR}$. This collection encompasses all NGBBs with yet-to-be-assigned active nodes within the partially reconstructed NOE graph and is crucial for the correct continuation of the methyl walk. The term $\:{p}_{active}$ denotes the probability of the active node having a specific amino acid type, and is given by Eq. 2:

2 $$\:{p_{active}} = \frac{{{n_{NGBB}}}}{{{n_{total}}}}$$

where $\:{n}_{NGBB}$ denotes the count of NGBBs in $\:{G}_{NMR}\:$having the same active node amino acid type as the NGBB under scrutiny and $\:{n}_{total}$ is the total amount of NGBBs in $\:{G}_{NMR}$. Finally, $\:{p}_{neighbor,i}$ determines the probability that a neighbouring node of the active node will exhibit a certain amino acid type, and as is given by Eq. 3:

3 $$\:{p_{neighbor,i}} = \frac{{{n_{neighbor,i}}}}{{{n_{total,neighbor}}}}$$

where $\:{n}_{total,neighbor}$ is the total number of neighboring nodes to an active node in $\:{G}_{NMR}$, and $\:{n}_{neighbor,i}$ is the count of neighboring nodes to active nodes in$\:{G}_{NMR}$ sharing the same amino acid type with the $\:i$-th neighbour of the active node in NGBB. Consequently, Eq. 3 calculates the probability that the $\:i$-th neighboring node of the active node has a certain amino acid type. Fig. S10 shows with an example how the *rarity score* is calculated. For seminal assignments, all unassigned NGBBs are part of $\:{G}_{NMR}$, as no partially reconstructed NOE graph exists at this point.

#### The similarity score

Similarity scores compare the local experimental NMR signature of a methyl group with the corresponding pattern predicted from the structural model. They consist of two components: a NOE term and an additional-restraints term (e.g., PCSs; Eq. 4), both of which quantify differences between NMR-derived and model-derived observations. The NOE term reflects the difference between the observed number of NOE contacts from a given methyl group to methyl groups of specific amino acid types and the corresponding number expected from the 3D structural model (Eq. 5).

The additional-restraints term reflects the difference between calculated and measured values for optional restraints such as PCSs (Eq. 6). If PCS data are unavailable for a given methyl group, for example because the shifts are too large to allow assignment or because the methyl group is too close to the paramagnetic centre, we instead use the difference to the mean value (Eq. 7). This ensures that missing additional-restraint data do not artificially favour such assignments.

More formally, the *similarity score* is used to identify the best-matching SGBB to a given NGBB sharing the same amino acid type at their respective active node. The active node at each methyl walk step is defined as the donor of one directional edge to other nodes within a building block. The *similarity score* reflects the congruence of edge patterns, adjacent amino acid type, and both experimental and theoretical additional restraints (e.g., PCS). The larger the score, the more similar SGBB and NGBB are. The *similarity score* is composed of an NOE-based term or *similarity score*_*NOE*_, and an additional restraints-based term or *similarity score*_*additional restraints*_ as defined in Eq. 4:

4 $$\eqalign{ & \:similarity\:score\left( {NGBB,SGBB} \right) = \cr & similarity\:scor{e_{NOE}} - \:similarity\:scor{e_{additional\:restraints}} \cr}$$

The NOE-based term is defined in Eq. 5 as follows:

5 $$\eqalign{ & \:similarity\:scor{e_{NOE}}(NGBB,SGBB) = \cr & 2c{W_{NOE}} - |c - {E_{structure}}| - |c - {E_{NOE}}| \cr}$$

with *c* being the number of common edges between the NGBB and SGBB building blocks, $\:{E}_{NOE}$ and $\:{E}_{structure}$ being the total number of edges in NGBB and SGBB, respectively, and $\:{W}_{NOE}$ being the weight factor for NOE-based restraints, which is typically set to 1 (for more details see supplementary methods).

The additional restraints-based term is only used when additional restraints are provided, and is defined in Eq. 6 as:

6 $$\eqalign{ & \:similarity\:scor{e_{additional\:restraints}}(NGBB,SGBB) = \cr & \sum \: _{i = 1}^4{W_i}|{a_{i,exp}} - {a_{i,theo}}|\: \cr} $$

where $\:{W}_{i}$ represents weight factor of the $\:i$-th set of additional restraints, and where $\:{a}_{i,exp}$ and $\:{a}_{i,theo}$ are experimentally and theoretically determined additional restraints associated with the active nodes of the NGBB and SGBB, respectively. This score supports up to four additional restraints ($\:i$ max = 4). If a particular $\:{a}_{i,exp}$ or $\:{a}_{i,theo}$ is not provided, the corresponding additional restraint-based term is calculated according to Eq. 7:

7 $$\eqalign{ & \:similarity\:scor{e_{additional\:restraints}}\left( {NGBB,SGBB} \right) = \cr & \sum \: _{i = 1}^410{W_i}\left| {\mu {\:_{i,exp}} - \mu {\:_{i,theo}}} \right|\:\:\:\: \cr}$$

with $\:{\mu\:}_{i,exp}$ and $\:{\mu\:}_{i,theo}$ being the mean of the absolute values of the other $\:i$-th experimentally or theoretically determined sets of additional restraints. Therefore, AMIGO considers the cases where no additional restraints are provided and is not biased towards assignments without additional restraints. This procedure has been empirically validated for all proteins presented in the benchmark test here. Fig. S11 illustrates an example of how the *similarity score*_*NOE*_ is calculated.

#### The total score

AMIGO generates an individual methyl walk for every NGBB, leading to an array of possible assignments as a function of the methyl walk starting point. Only the methyl walk with the largest *total score*, will be provided by AMIGO as the correct assignment. The *total score* is calculated according to Eq. 8 as:

8 $$\eqalign{ & \:total\:score\left( {{A_{methyl\:walk}}} \right) = \cr & \sum \: _{i = 1}^Nsimilarity\:score(NGB{B_i},SGB{B_{,i}})\: \cr}$$

with $\:{A}_{methyl\:walk}$ being a set of *N* building block pairs $\:(NGB{B}_{i},\:SGB{B}_{i})$, whose active nodes have been assigned to each other in a given methyl walk.

## Supplementary Information

Below is the link to the electronic supplementary material.

Supplementary Material 1

## Data Availability

See code availability.

## Code Availability

*Python* scripts used for data analysis can be downloaded free of charge from https://github.com/ThorbenMaa/AMIGO.

## Declarations

### Parameter Optimization

A detailed explanation on parameter optimization required to run AMIGO can be found in the corresponding chapter in the SI, p. 13.

## References

[CR1] Amero C, Asunción Durá M, Noirclerc-Savoye M et al (2011) A systematic mutagenesis-driven strategy for site-resolved NMR studies of supramolecular assemblies. J Biomol NMR 50:229–236. 10.1007/s10858-011-9513-5 21626214 10.1007/s10858-011-9513-5

[CR2] Baldwin AJ, Kay LE (2009) NMR spectroscopy brings invisible protein states into focus. Nat Chem Biol 5:808–814. 10.1038/nchembio.238 19841630 10.1038/nchembio.238

[CR3] Barrett PJ, Chen J, Cho M-K et al (2013) The quiet renaissance of protein nuclear magnetic resonance. Biochemistry 52:1303–1320. 10.1021/bi4000436 23368985 10.1021/bi4000436 PMC3592982

[CR4] Bax A, Clore GM (2019) Protein NMR: boundless opportunities. J Magn Reson 306:187–191. 10.1016/j.jmr.2019.07.037 31311710 10.1016/j.jmr.2019.07.037 PMC6703950

[CR6] Chao F-A, Kim J, Xia Y et al (2014) FLAMEnGO 2.0: an enhanced fuzzy logic algorithm for structure-based assignment of methyl group resonances. J Magn Reson 245:17–23. 10.1016/j.jmr.2014.04.012 24915505 10.1016/j.jmr.2014.04.012 PMC4161213

[CR5] Chao F-A, Shi L, Masterson LR, Veglia G (2012) FLAMEnGO: a fuzzy logic approach for methyl group assignment using NOESY and paramagnetic relaxation enhancement data. J Magn Reson 214:103–110. 10.1016/j.jmr.2011.10.008 22134225 10.1016/j.jmr.2011.10.008 PMC3487468

[CR7] Clay MC, Saleh T, Kamatham S et al (2022) Progress toward automated methyl assignments for methyl-TROSY applications. Structure 30:69-79.e2. 10.1016/j.str.2021.11.009 34914892 10.1016/j.str.2021.11.009 PMC8741727

[CR45] Cordella LP, Foggia P, Sansone C, Vento M (2004) A (sub)graph isomorphism algorithm for matching large graphs. IEEE Trans Pattern Anal Mach Intell 26:1367–1372. 10.1109/TPAMI.2004.75 15641723 10.1109/TPAMI.2004.75

[CR49] Fiser A, Sali A (2003) ModLoop: automated modeling of loops in protein structures. Bioinformatics19:2500–2501. 10.1093/bioinformatics/btg362 10.1093/bioinformatics/btg362 14668246

[CR9] Flügge F, Peters T (2018) Complete assignment of Ala, Ile, Leu, Met and Val methyl groups of human blood group A and B glycosyltransferases using lanthanide-induced pseudocontact shifts and methyl–methyl NOESY. J Biomol NMR Assign 70:245–259. 10.1007/s10858-018-0183-4 10.1007/s10858-018-0183-4 29700756

[CR10] Gans P, Hamelin O, Sounier R et al (2010) Stereospecific Isotopic Labeling of Methyl Groups for NMR Spectroscopic Studies of High‐Molecular‐Weight Proteins. Angew Chem Int Ed 49:1958–1962. 10.1002/anie.200905660 10.1002/anie.200905660 20157899

[CR11] Gauto DF, Estrozi LF, Schwieters CD et al (2019) Integrated NMR and cryo-EM atomic-resolution structure determination of a half-megadalton enzyme complex. Nat Commun 10:2697. 10.1038/s41467-019-10490-9 31217444 10.1038/s41467-019-10490-9 PMC6584647

[CR12] John M, Schmitz C, Park AY et al (2007) Sequence-specific and stereospecific assignment of methyl groups using paramagnetic lanthanides. J Am Chem Soc 129:13749–13757. 10.1021/ja0744753 17929923 10.1021/ja0744753

[CR13] Karamanos TK, Matthews S (2023) Biomolecular NMR in the AI-assisted structural biology era: Old tricks and new opportunities. Biochimica et Biophysica Acta (BBA) - Proteins and Proteomics 140949. 10.1016/j.bbapap.2023.140949 10.1016/j.bbapap.2023.140949 37572958

[CR14] Klukowski P, Riek R, Güntert P (2022) Rapid protein assignments and structures from raw NMR spectra with the deep learning technique ARTINA. Nat Commun 13:6151. 10.1038/s41467-022-33879-5 36257955 10.1038/s41467-022-33879-5 PMC9579175

[CR15] Krempl C, Wurm JP, Beck Erlach M et al (2023) Insights into the structure of invisible conformations of large methyl group labelled molecular machines from high pressure NMR. J Mol Biol 435:167922. 10.1016/j.jmb.2022.167922 37330282 10.1016/j.jmb.2022.167922

[CR16] Lescanne M, Skinner SP, Blok A et al (2017) Methyl group assignment using pseudocontact shifts with PARAssign. J Biomol NMR Assign 69:183–195. 10.1007/s10858-017-0136-3 10.1007/s10858-017-0136-3 PMC5736784 29181729

[CR17] Maass T, Westermann LT, Creutznacher R et al (2022) Assignment of Ala, Ile, LeuproS, Met, and ValproS methyl groups of the protruding domain of murine norovirus capsid protein VP1 using methyl–methyl NOEs, site directed mutagenesis, and pseudocontact shifts. Biomol NMR Assign 16:97–107. 10.1007/s12104-022-10066-7 35050443 10.1007/s12104-022-10066-7 PMC9068638

[CR18] Mas G, Guan J-Y, Crublet E et al (2018) Structural investigation of a chaperonin in action reveals how nucleotide binding regulates the functional cycle. Sci Adv 4:eaau4196. 10.1126/sciadv.aau4196 30255156 10.1126/sciadv.aau4196 PMC6154984

[CR46] McGregor JJ (1982) Backtrack search algorithms and the maximal common subgraph problem. Softw Pract Exp 12:23–34. 10.1002/spe.4380120103

[CR19] McShan AC (2023) Utility of methyl side chain probes for solution NMR studies of large proteins. J Magn Reson Open 14:100087. 10.1016/j.jmro.2022.100087

[CR22] Mühlberg L, Alarcin T, Maass T et al (2022) Ligand-induced structural transitions combined with paramagnetic ions facilitate unambiguous NMR assignments of methyl groups in large proteins. J Biomol NMR 76:59–74. 10.1007/s10858-022-00394-0 35397749 10.1007/s10858-022-00394-0 PMC9247001

[CR20] Mohanty B, Orts J, Wang G et al (2022) Methyl probes in proteins for determining ligand binding mode in weak protein–ligand complexes. Sci Rep 12:11231. 10.1038/s41598-022-13561-y 35789157 10.1038/s41598-022-13561-y PMC9253027

[CR21] Monneau YR, Rossi P, Bhaumik A et al (2017) Automatic methyl assignment in large proteins by the MAGIC algorithm. J Biomol NMR 69:215–227. 10.1007/s10858-017-0149-y 29098507 10.1007/s10858-017-0149-y PMC5764113

[CR23] Nerli S, De Paula VS, McShan AC, Sgourakis NG (2021) Backbone-independent NMR resonance assignments of methyl probes in large proteins. Nat Commun 12:691. 10.1038/s41467-021-20984-0 33514730 10.1038/s41467-021-20984-0 PMC7846771

[CR24] Neuhaus D (2011) Nuclear Overhauser Effect. In: Harris RK (ed) Encyclopedia of Magnetic Resonance. John Wiley & Sons, Ltd, Chichester, UK, p emrstm0350pub2

[CR48] Orton HW, Huber T, Otting G (2020) Paramagpy: software for fitting magnetic susceptibilitytensors using paramagnetic effects measured in NMR spectra. Magn Reson 1:1–12. 10.5194/mr-1-1-2020 10.5194/mr-1-1-2020 PMC10500712 37904891

[CR25] Orts J, Vögeli B, Riek R (2012) Relaxation matrix analysis of spin diffusion for the NMR structure calculation with eNOEs. J Chem Theory Comput 8:3483–3492. 10.1021/ct3002249 26592998 10.1021/ct3002249

[CR26] Otting G (2010) Protein NMR using paramagnetic ions. Annu Rev Biophys 39:387–405. 10.1146/annurev.biophys.093008.131321 20462377 10.1146/annurev.biophys.093008.131321

[CR27] Pederson K, Chalmers GR, Gao Q et al (2017) <article-title update="added">NMR characterization of HtpG, the *E. coli* Hsp90, using sparse labeling with ^13^C-methyl alanine. J Biomol NMR 68(3):225–236. 10.1007/s10858-017-0123-8 28653216 10.1007/s10858-017-0123-8 PMC5546222

[CR30] Pritišanac I, Alderson TR, Güntert P (2020) Automated assignment of methyl NMR spectra from large proteins. Prog Nucl Magn Reson Spectrosc 118–119:54–73. 10.1016/j.pnmrs.2020.04.001 10.1016/j.pnmrs.2020.04.001 32883449

[CR28] Pritišanac I, Degiacomi MT, Alderson TR et al (2017) Automatic assignment of methyl-NMR spectra of supramolecular machines using graph theory. J Am Chem Soc 139:9523–9533. 10.1021/jacs.6b11358 28691806 10.1021/jacs.6b11358

[CR29] Pritišanac I, Würz JM, Alderson TR, Güntert P (2019) Automatic structure-based NMR methyl resonance assignment in large proteins. Nat Commun 10:4922. 10.1038/s41467-019-12837-8 31664028 10.1038/s41467-019-12837-8 PMC6820720

[CR32] Proudfoot A, Frank AO, Frommlet A, Lingel A (2019) Selective Methyl Labelling of Proteins: Enabling Structural and Mechanistic Studies As Well As Drug Discovery Applications by Solution-State NMR. Methods in Enzymology. Elsevier, pp 1–36 10.1016/bs.mie.2018.08.035 30611421

[CR31] Proudfoot A, Frank AO, Ruggiu F et al (2016) Facilitating unambiguous NMR assignments and enabling higher probe density through selective labelling of all methyl containing amino acids. J Biomol NMR 65:15–27. 10.1007/s10858-016-0032-2 27130242 10.1007/s10858-016-0032-2

[CR33] Rossi P, Monneau YR, Xia Y et al (2019) Toolkit for NMR Studies of Methyl-Labelled Proteins. Methods in Enzymology. Elsevier, pp 107–142 10.1016/bs.mie.2018.08.036 30611422

[CR34] Schütz S, Sprangers R (2020) Methyl TROSY spectroscopy: a versatile NMR approach to study challenging biological systems. Prog Nucl Magn Reson Spectrosc 116:56–84. 10.1016/j.pnmrs.2019.09.004 32130959 10.1016/j.pnmrs.2019.09.004

[CR35] Shiraishi Y, Natsume M, Kofuku Y et al (2018) Phosphorylation-induced conformation of β2-adrenoceptor related to arrestin recruitment revealed by NMR. Nat Commun 9:194. 10.1038/s41467-017-02632-8 29335412 10.1038/s41467-017-02632-8 PMC5768704

[CR47] Skinner SP, Fogh RH, Boucher W et al (2016) CcpNmr AnalysisAssign: a flexible platform forintegrated NMR analysis. J Biomol NMR 66:111–124. 10.1007/s10858-016-0060-y 27663422 10.1007/s10858-016-0060-y PMC5095159

[CR37] Sprangers R, Gribun A, Hwang PM et al (2005) Quantitative NMR spectroscopy of supramolecular complexes: dynamic side pores in ClpP are important for product release. Proc Natl Acad Sci USA 102:16678–16683. 10.1073/pnas.0507370102 16263929 10.1073/pnas.0507370102 PMC1283831

[CR36] Sprangers R, Kay LE (2007) Quantitative dynamics and binding studies of the 20S proteasome by NMR. Nature 445:618–622. 10.1038/nature05512 17237764 10.1038/nature05512

[CR38] Tugarinov V, Hwang PM, Ollerenshaw JE, Kay LE (2003) Cross-correlated relaxation enhanced ^1^ H – ^13^ C NMR spectroscopy of methyl groups in very high molecular weight proteins and protein complexes. J Am Chem Soc 125:10420–10428. 10.1021/ja030153x 12926967 10.1021/ja030153x

[CR39] Tugarinov V, Kay LE, Ibraghimov I, Orekhov VY (2005) High-resolution four-dimensional ^1^ H – ^13^ C NOE spectroscopy using methyl-TROSY, sparse data acquisition, and multidimensional decomposition. J Am Chem Soc 127:2767–2775. 10.1021/ja044032o 15725035 10.1021/ja044032o

[CR40] Venditti V, Fawzi NL, Clore GM (2011) Automated sequence- and stereo-specific assignment of methyl-labelled proteins by paramagnetic relaxation and methyl–methyl nuclear overhauser enhancement spectroscopy. J Biomol NMR 51:319–328. 10.1007/s10858-011-9559-4 21935714 10.1007/s10858-011-9559-4 PMC3212433

[CR41] Wen J, Zhou P, Wu J (2012) Efficient acquisition of high-resolution 4-D diagonal-suppressed methyl–methyl NOESY for large proteins. J Magn Reson 218:128–132. 10.1016/j.jmr.2012.02.021 22464875 10.1016/j.jmr.2012.02.021 PMC3625671

[CR43] Williamson MP (2013) Using chemical shift perturbation to characterise ligand binding. Prog Nucl Magn Reson Spectrosc 73:1–16. 10.1016/j.pnmrs.2013.02.001 23962882 10.1016/j.pnmrs.2013.02.001

[CR42] Williams RV, Rogals MJ, Eletsky A et al (2022) AssignSLP_GUI, a software tool exploiting AI for NMR resonance assignment of sparsely labelled proteins. J Magn Reson 345:107336. 10.1016/j.jmr.2022.107336 36442299 10.1016/j.jmr.2022.107336 PMC9742323

[CR44] Xu Y, Matthews S (2013) Map-xsii: an improved program for the automatic assignment of methyl resonances in large proteins. J Biomol NMR 55:179–187. 10.1007/s10858-012-9700-z 23292498 10.1007/s10858-012-9700-z

