# Supplementary material for: AMIGO - Guided assignment of 13C-methyl labelled proteins
Source: J Biomol NMR. 2026 Apr 13;80(1):12. doi: 10.1007/s10858-026-00491-4 (PMC13070982; doi:10.1007/s10858-026-00491-4)
Supplement: Supplementary file 1 — Supplementary Material 1 [file 10858_2026_491_MOESM1_ESM.docx]

**AMIGO - Guided Assignment of ^13^C-Methyl Labelled Proteins**

Thorben Maass^1^, Lorena Rudolph^1^, Thomas Peters,^2^ and Alvaro Mallagaray^1^*

^1^Institute for Chemistry and Metabolomics, Centre for Structural and Cell Biology in Medicine, University of Lübeck, 23562 Lübeck, Germany

^2^Institute of Biochemistry, Centre for Structural and Cell Biology in Medicine, University of Lübeck, 23562 Lübeck, Germany

Corresponding author: Alvaro Mallagaray. Email: alvaro.mallagaraydebenito@uni-luebeck.de

ORCID numbers:

Alvaro Mallagaray: 0000-0001-5825-4407

Thorben Maass: 0000-0003-4764-2740

Thomas Peters: 0000-0002-7570-8260

Lorena Rudolph: [0000-0002-0218-2747](https://orcid.org/0000-0002-0218-2747)

**Table of Contents:**

1. Input files required by AMIGO 3

2. How to adjust parameters and run AMIGO 6

2.1 Running the interactive version of AMIGO 6

3. Outputs generated by AMIGO to guide the assignment process 7

3.1 Text file results.txt 7

3.2 Methyl walk graphs on the NOE spectrum and on the structural model(s) 8

Fig. S1 Complete NOE-based graph observed for the protein Lm-UGP showing the methyl walks 9

Fig. S2 Structure-based graph created for the protein Lm-UGP showing the methyl walks 10

3.3 Validation of assignment using experimentally and theoretically determined additional restraints 11

Fig. S3 Example for assignment validation using experimentally and theoretically determined additional restraints (PCSs) for the LmUGP data set. 11

Parameter optimisation 12

Structural ensembles 12

How SGGBs can be combined to reconstruct any structure-based graph 13

Fig S4 Any possible structure graph can be created from a linear combination of structure graph building blocks (SGBB) 13

Constructing methyl walks from graph building blocks (GBBs) – a detailed explanation 13

Fig S5 Step 1 of the identification of methyl walks from graph building blocks 14

Fig S6 Step 2 - assignment of the first resonance 14

Fig S7 Step 3 of the identification of methyl walks from graph building blocks 15

Fig S8 Step 4 of the identification of methyl walks from graph building blocks 16

Fig S9 The methyl walk advances by iteration from Step 2 16

Examples illustrating the calculation of *similarity* and *rarity scores* 17

Fig S10 Example of calculation of the *rarity score* 17

Fig S11 Exemplary calculation of the *similarity score_NOE_*. 17

About the assignment of methyl groups in close proximity to paramagnetic metals 18

Fig S12 Test runs for the determination of optimal range cut-off distances for the benchmark set in the absence of additional restraints 20

Table S1 Reference assignments for the tested experimental data sets 21

Table S2 Proteins used to create PDB-based synthetic NOE networks 21

Bibliography 22

## 1. Input files required by AMIGO

All files described in this manuscript are freely available and can be downloaded from the *github* repository following the link: <https://github.com/ThorbenMaa/AMIGO>.

AMIGO is implemented in *python3* and has been successfully tested under Linux and Mac (Sonoma 14.1.1) environments. The use of AMIGO is illustrated in this guide with the assignment of two proteins, GTB (Flügge and Peters 2018) and LmUGP (Mühlberg et al. 2022). The protein GTB has been used to illustrate chapters 1 & 2, and the corresponding files can be found in *github* under *InputFilesExample_GTB*. Chapter 3 is illustrated with the outcome produced during the assignment of the protein LmUGP. Corresponding files can be found in *github* under *InputFilesExample_LmUGP*. These examples have been selected to best illustrate AMIGO’s capabilities. Additionally, all input and output files from the assignment of the Benchmark proteins can be found under *Benchmark*.

After setting up the environment necessary for AMIGO as described in our *github* repository, place all necessary files in a single folder.


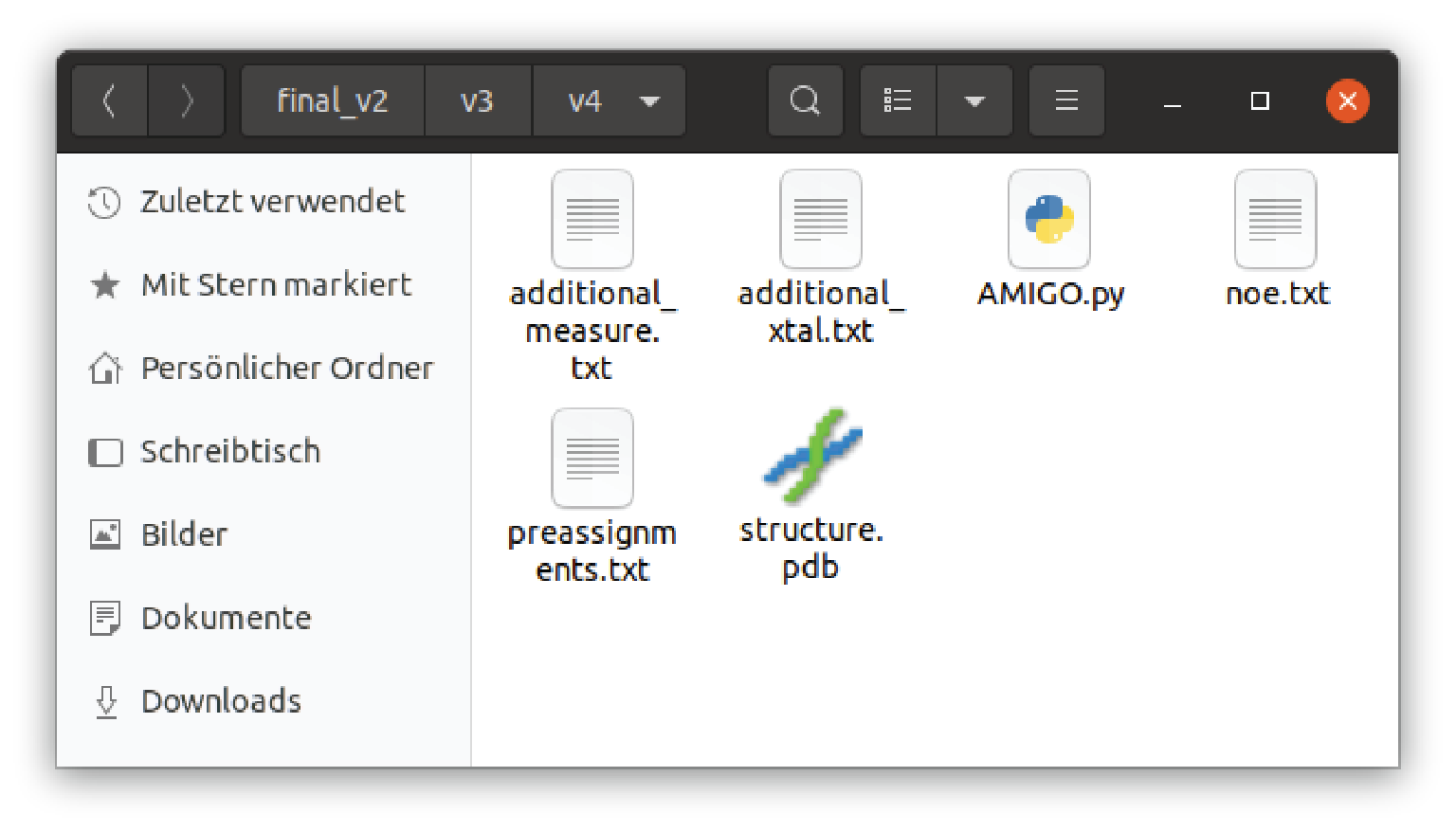


The file noe.txt is a text file organized as follows: The first column contains the amnio acid type of the auto peak in a multidimensional NOESY experiment (e.g. 4d-HMQC-NOESY-HMQC), while the second column corresponds to an arbitrary peak ID, which is typically an integer. Each methyl group receives an unique ID number. The third and fourth columns contain the amino acid type and peak ID of the NOESY cross-peaks observed in the NOESY plane corresponding to the frequencies of the selected auto peak. The list must be sorted using the second column to allow AMIGO to create “NOE graph-based building blocks” (see results). E.g., sorting can be easily done using Excel. Columns are tab separated. AMIGO gives an error message if the last line of this file is empty, so there must not be a line break at the end of the list. See figure below for an example:


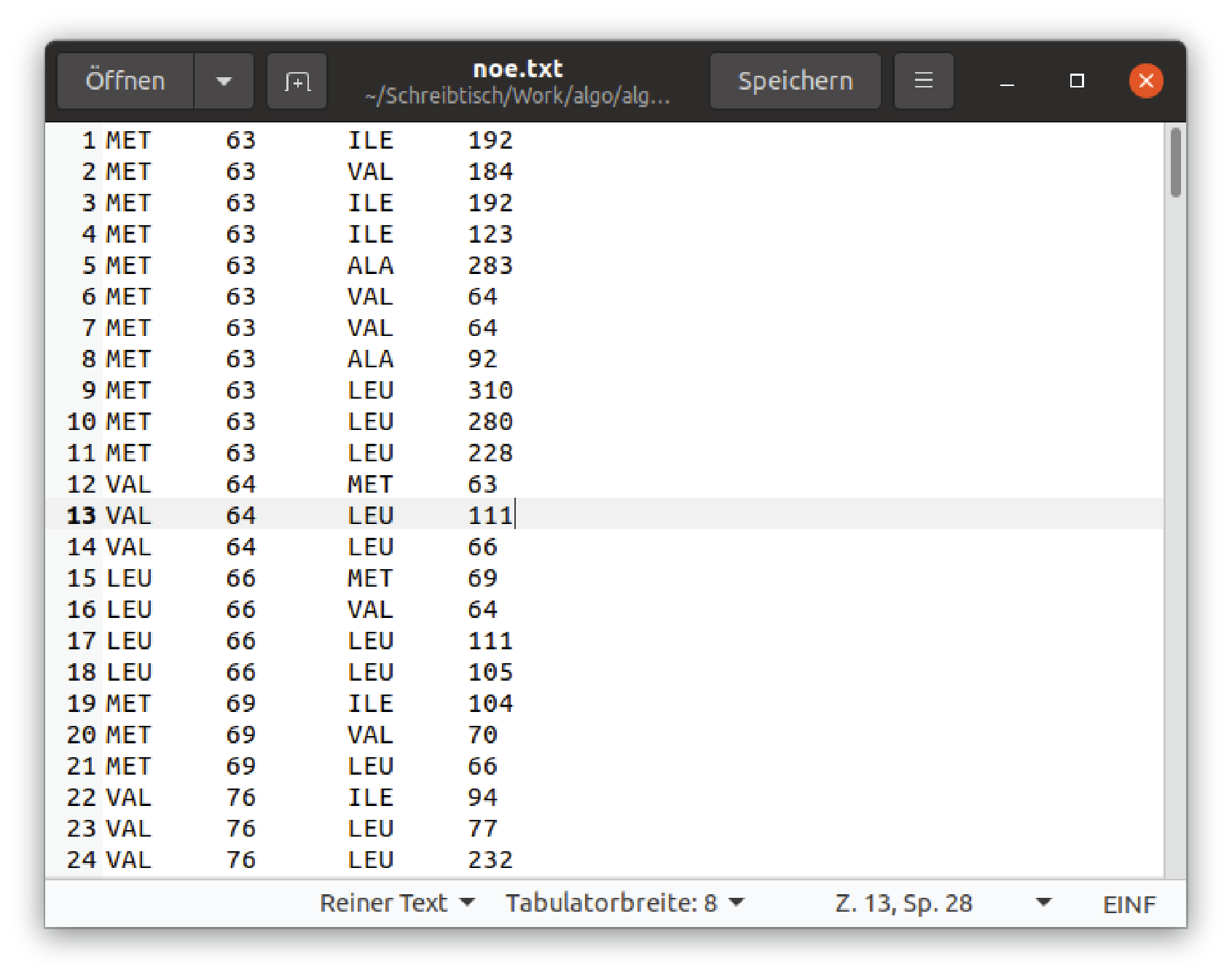


The file additional_measure.txt is a text file containing additional experimental restraints (e.g. measured PCSs). The first column contains the amino acid type of a certain methyl group resonance. The second column contains the peak ID, which needs to match with that form the noe.txt list. The third, fourth, fifth and sixth columns contain the respective additional experimental restraints. In the example below, column three contains PCSs induced by Ce^3+^, column four PCSs induced by Eu^3+^, column five PCSs induced by Tb^3+^ and column six corresponds to no data, therefore containing the number 999. Use also 999 within a list of additional restraints if a specific methyl group lacks the information (e.g. the peaks in the spectrum were not visible or superimposed). If no additional restraints are available at all, simply use a single line containing: “0 0 0 0 0 0” as the only file content. Columns are tab separated.


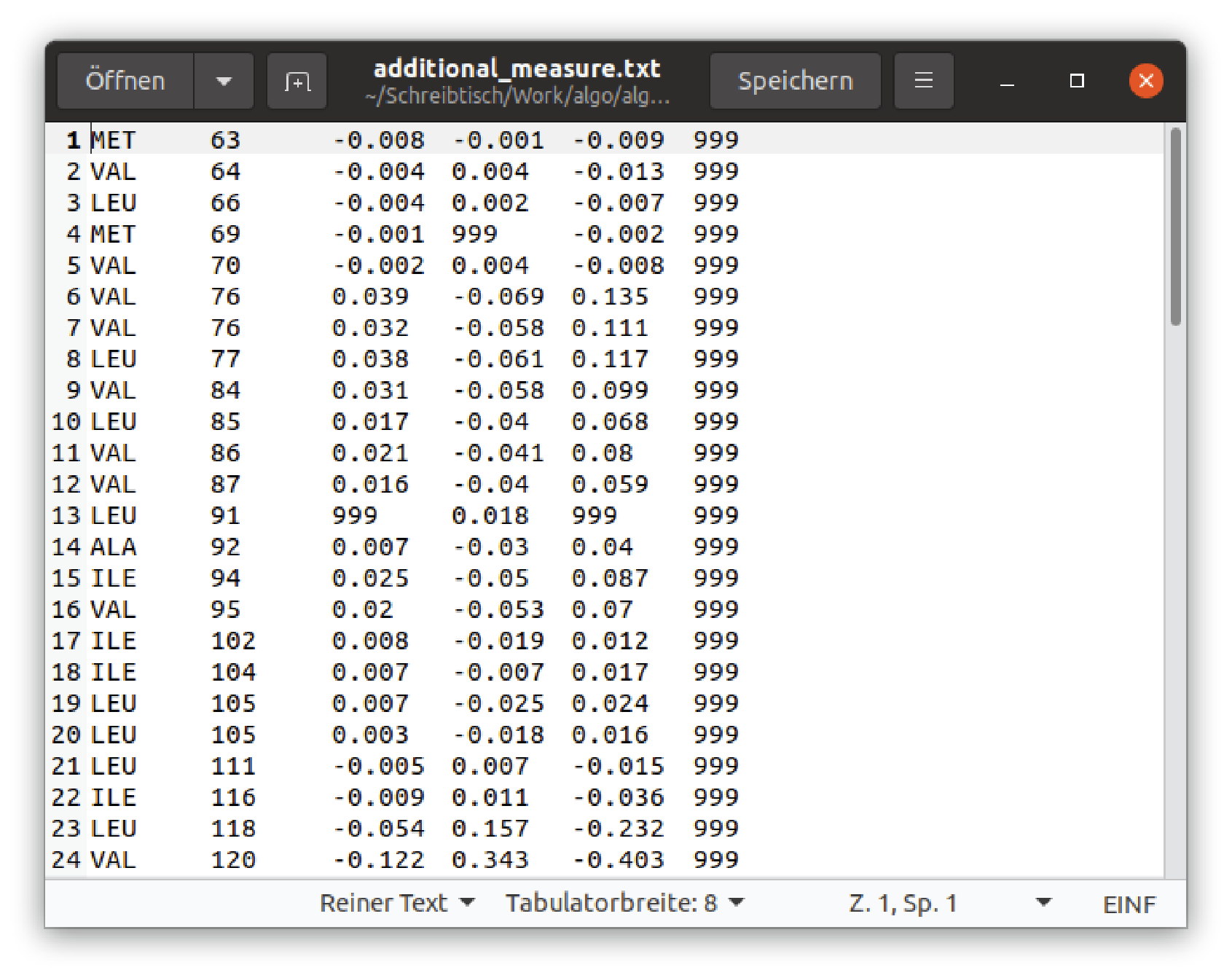


The file additional_xtal.txt is a text file containing theoretically determined additional restraints according to the structural model (crystal structure) provided. The first and second columns contain the amino acid type and the residue number in the protein sequence, respectively. Columns three, four, five, and six contain theoretically calculated additional restraints (eg. PCS). The order of the additional restraints must match the order in additional_measure.txt. Use 999 like in additional_measure.txt file. Likewise, if no theoretically determined additional restraints are available at all, simply use a single line containing: “0 0 0 0 0 0” as the only file content. Columns are tab separated.


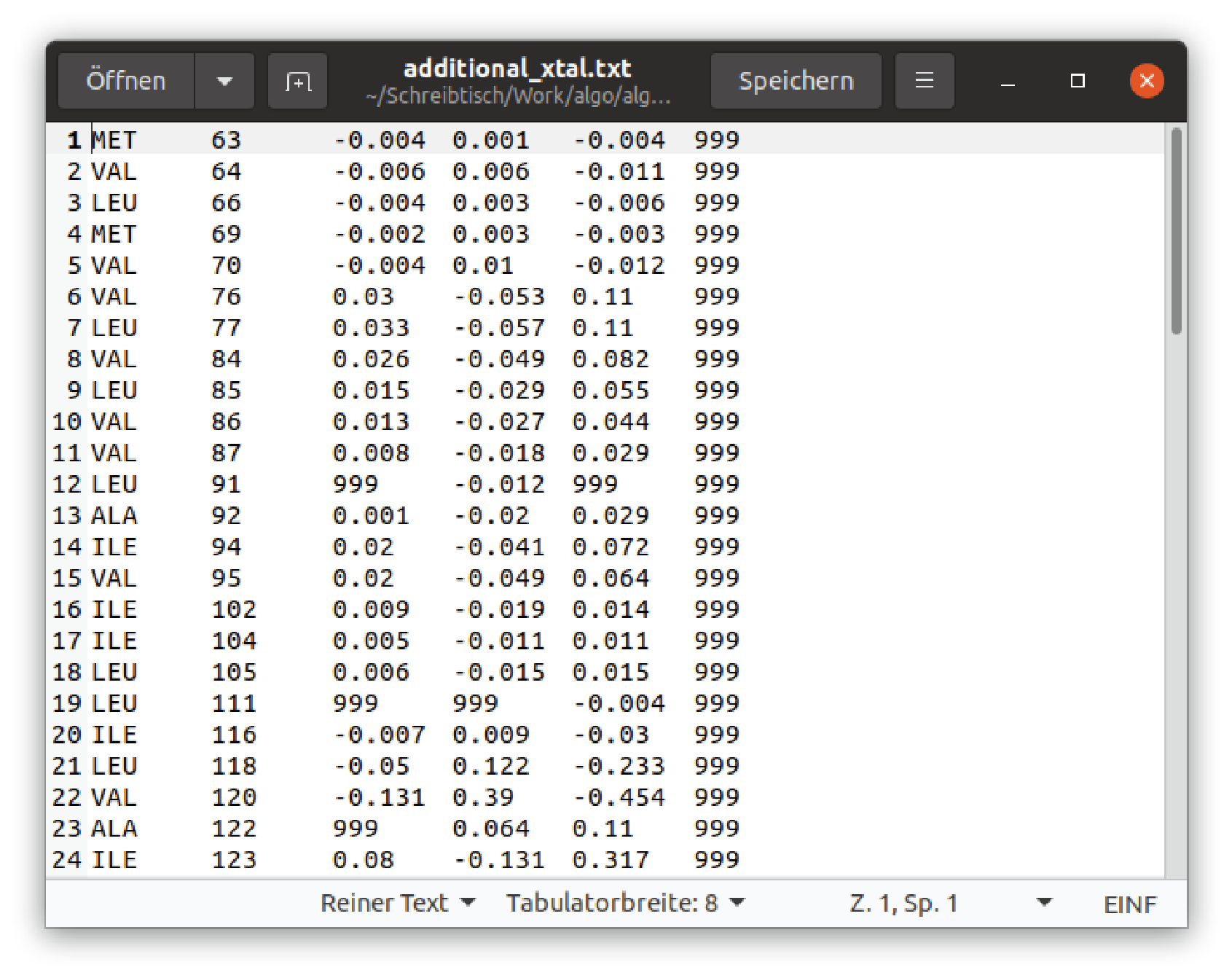


The file preassignments.txt is a text file where the first and second columns contain the amino acid type and peak ID of a certain methyl group resonance, respectively. The peak ID must match with those in previous files. The third and fourth columns contain the amino acid type and residue number of the methyl group from the protein sequence in the crystal structure the resonance is assigned to. For an example see the figure below. If no pre-assignments are available, just leave this text file empty.

When pre-assignments are included in the calculations, AMIGO will in a first step consider only the pre-assigned resonances as starting points to create the methyl walks. Afterwards, all remaining methyl group resonances are considered.


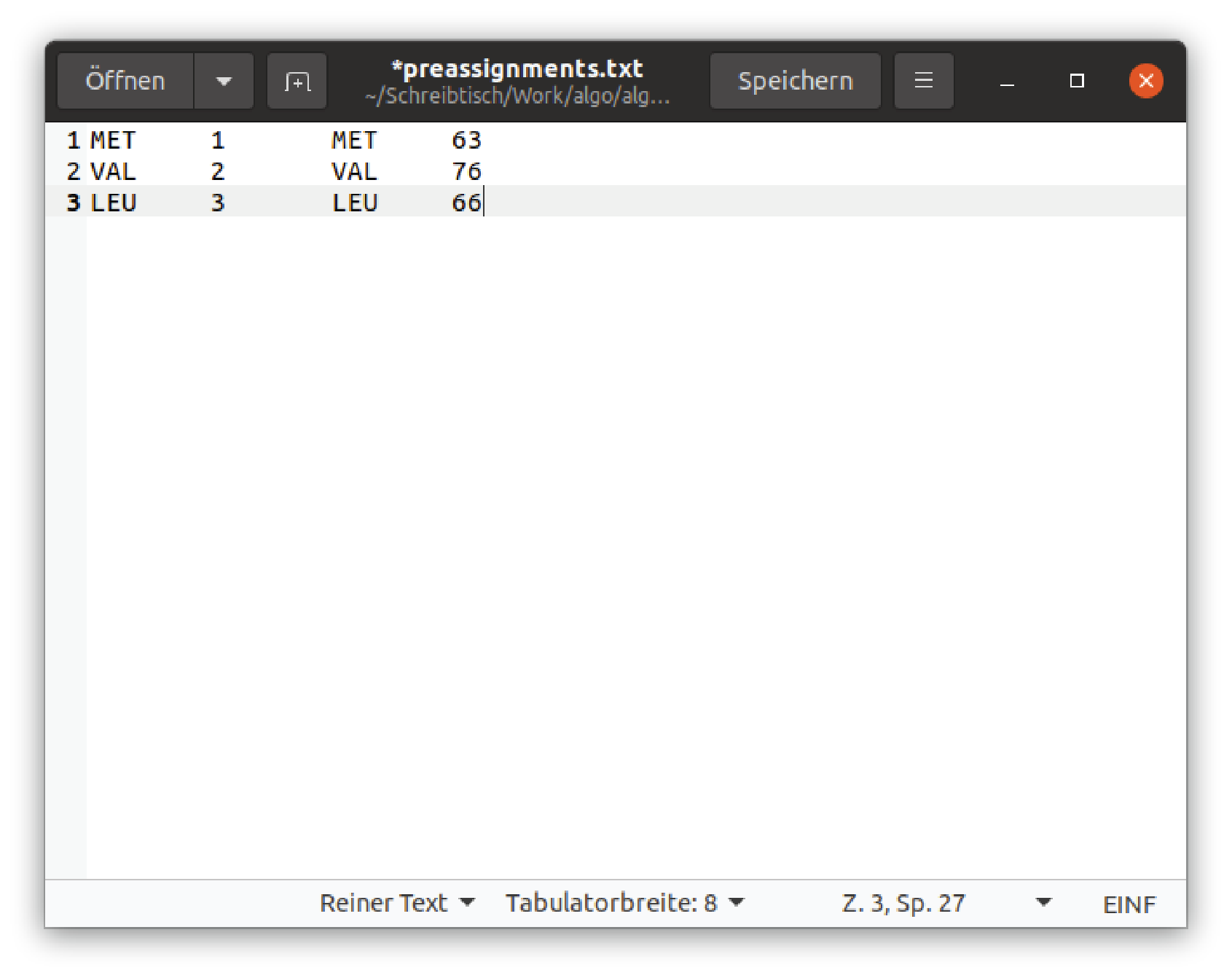


Finally, the structure.pdb file is just a text pdb file containing the atom coordinates of the protein to be assigned. To note, structural ensembles can also be provided to AMIGO. To do so, align the structures and provide the coordinates in a single .pdb file. The NOEs of the two or more structures need to be submitted as one single NOE list file.

Upon completion of the assignments, AMIGO will produce a filed named results.txt within the same folder containing the assignments proposed and the methyl walks followed to obtain such assignments.

## 2. How to adjust parameters and run AMIGO

AMIGO can be executed in using the file *run_AMIGO.sh*. Typing **bash run_AMIGO.sh** will execute *AMIGO_non_interactive.py*, which reads the parameters from *run_AMIGO.sh* and runs AMIGO. The parameters are explained in the following.

- 1. **Running the interactive version of AMIGO**

AMIGO needs several input parameters as explained in the corresponding tutorials provided on *github*. One of them is the weighting factor $W_{NOE}$ in Eq. 2. The recommended value is 1.

Additionally, AMIGO inquires the weight factors of the additional parameter restraints $W_{i}$ (Eq. 3), with $i$ being 1 to 4. A value of 0 indicates that for that specific $W_{i}$ no data is available. Any other value indicates the presence of data. When pseudo contact shifts (PCS) are provided we recommend setting $W_{i}$ to 100. Long range structural data corresponding to paramagnetic relaxation enhancements (PRE) have not been tested within the benchmark, but they can be used like PCS.

Next, AMIGO allows to define an amino acid specific minimum and maximum distance cut-off between methyl groups to be explored when constructing the structural model building blocks. The size of each incremental step (*distance step range*) is also required. Typical values are 0.1 or 0.2 for optimal compromise between computational time and number of correct assignments. Higher confidence in the assignments can be obtained with values below 0.1, although at the cost of computation time. All distances are given in Å and with a single decimal. In case a certain amino acid type has not been isotopically labelled, the value 0 is to be used as minimum and maximum cut-off distances. In this case the distance step range = 1.

The last parameter required for running AMIGO is the number of potential starting points that should be considered. The value 1 would be corresponding to 100% of the methyl groups. Lower values can be used to save calculation time (e.g., 0.1 for only using the 10% most unique methyl groups), at the risk of a small increase in the number of unassigned methyl groups.

AMIGO will show a progress count indicating the extent of the assignment completed after each iteration, allowing the user to estimate the time required until completion.

A full example including all parameters is given below:

#!/bin/bash

python AMIGO_non_interactive.py \

--pdb_file 4m2a_pro.pdb \

--noe_file noe.txt \

--additional_measure_file additional_measure.txt \

--additional_xtal_file additional_xtal.txt \

--preassignments_file preassignments.txt \

--min_met 3 \

--max_met 9 \

--step_met 0.2 \

--min_ile 3 \

--max_ile 9 \

--step_ile 0.2 \

--min_leu 3 \

--max_leu 9 \

--step_leu 0.2 \

--min_val 3 \

--max_val 9 \

--step_val 0.2 \

--min_ala 3 \

--max_ala 9 \

--step_ala 0.2 \

--min_thr 3 \

--max_thr 9 \

--step_thr 0.2 \

--weight_noe 1 \

--weight_additional_resraint1 0 \

--weight_additional_resraint2 0 \

--weight_additional_resraint3 0 \

--weight_additional_resraint4 0 \

--percentage_starting_points 1 \

--val_scheme proS \

--leu_scheme proS

use “proS” “proR” or “both” for the Leu and Val labeling schemes.

For more information and complete tutorials see files and examples located in <https://github.com/ThorbenMaa/AMIGO>.

## 3. Outputs generated by AMIGO to guide the assignment process

**3.1 Text file results.txt**

AMIGO will generate a file results.txt containing a list of the assignments, step-by-step methyl walks and other complementary data that can be used to evaluate the goodness of the assignment. We will illustrate the typical results produced by AMIGO with one example belonging to the assignment of LmUGP with the inclusion of CSPs, and using a cut-off distance range of 3 to 9.5 Å. The complete file can be found as part of the tested protein benchmark. This example illustrates the assignment of Leu 204 from the active node Leu 317. Leu 204 is the last amino acid assigned within a methyl walk started in Leu 107. The complete methyl walk up to the current active node is indicated by AMIGO as follows:

methyl walk (NMR//pdb) : start => LEU 107 // LEU 107 => THR 139 // THR 139 => LEU 129 // LEU 129 => THR 110 // THR 110 => VAL 114 // VAL 114 => VAL 78 // VAL 78 => THR 76 // THR 76 => LEU 232 // LEU 232 => MET 235 // MET 235 => ILE 312 // ILE 312 => MET 215 // MET 215 => MET 128 // MET 128 => VAL 77 // VAL 77 => LEU 314 // LEU 314 => LEU 317 // LEU 317

For every step in every methyl walk AMIGO shows all previous steps followed from the seminal assignment (Leu 107) to the active node (Leu 317). AMIGO will then display the current step in the methyl walk, showing the active node (Leu 317) and the next amino acid assigned in this methyl walk (Leu 204). It also shows that the NOE between Leu 204 and Leu 317 is generated by methyl groups located within 3.8 Å apart from each other, according to the crystal structure. Such information can be used to evaluate the goodness of the assignment. Assignments obtained through long-range NOEs are normally indicative of a low confidence assignment:

was extended with methyl group resonance: LEU 204 with NOEs to resonances LEU 317 /

and thereby assigned to pdb methyl group: LEU 204 with expected NOEs to LEU 317 / applying a cut-off distance of 3.8000000000000003 Angstroms

The next two lines show that the experimentally obtained and theoretically determined additional restraints are in very well agreement, further reassuring the assignment. Note that the value “999” is a placeholder for not determined values:

additional experimental restraints are [additional restraint 1, 2, 3 and 4]: [999.0, 0.021, 0.008, 999.0]

additional theoretical restraints are [additional restraint 1, 2, 3 and 4]: [999.0, 0.02028, 0.00536, 999.0]

**3.2 Methyl walk graphs on the NOE spectrum and on the structural model(s)**

NOE-based (Figure S1) and structure-based (Figure S2) graphs are also created as part of the AMIGO results. They depict the path followed by AMIGO during the methyl walks. The nodes are colour coded with the amino acid type and annotated with the amino acid type and number. Edges indicate from which amino acid to which amino acid AMIGO jumped during the methyl walk. These graphs can be dynamically explored by the user and allow an intuitive assessment of the path followed by AMIGO during the assignment in the NOESY spectrum and in the crystal structure(s).


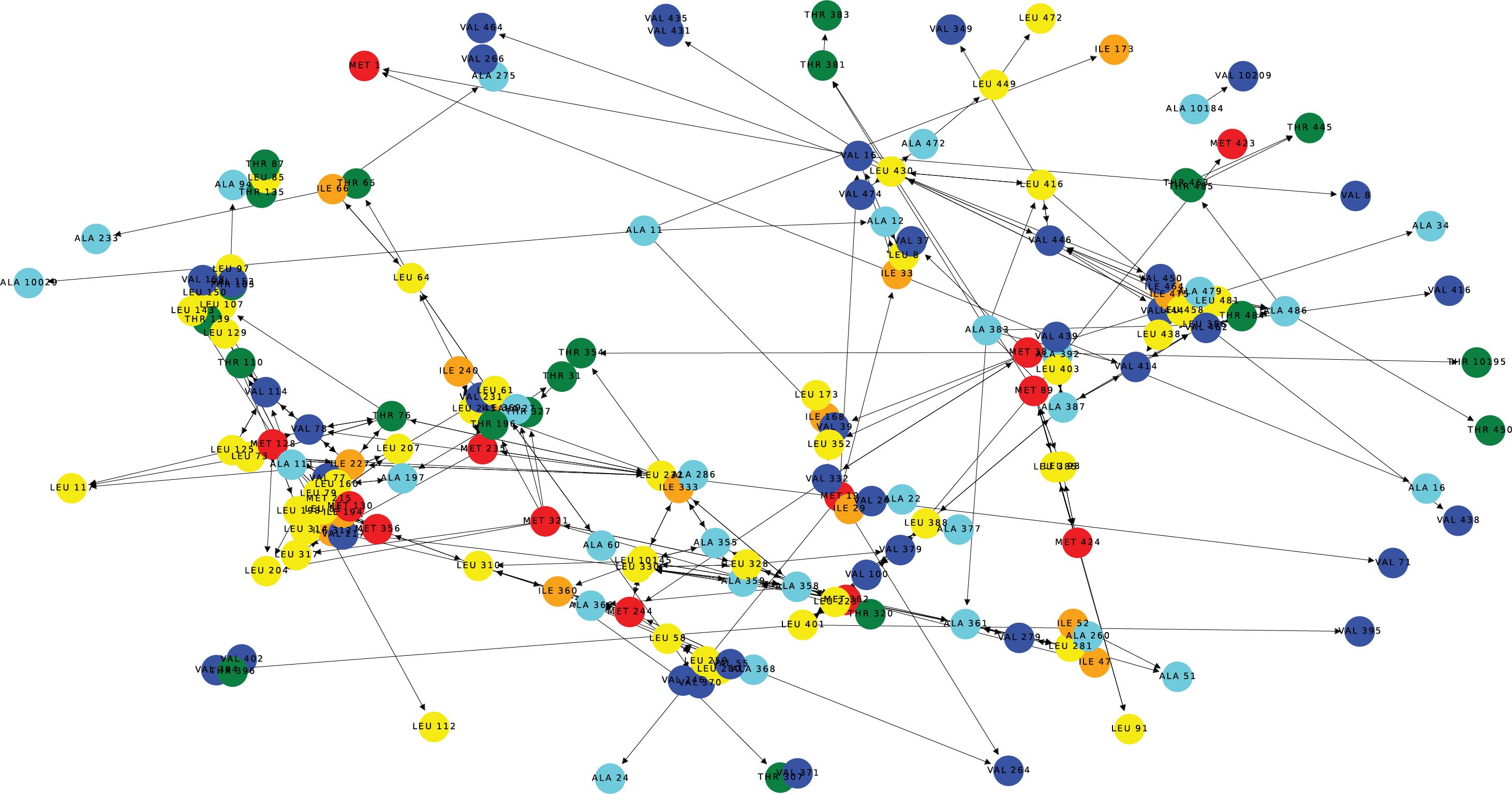


**Fig. S1 Complete NOE-based graph observed for the protein Lm-UGP showing the methyl walks.** Amino acids are colour-coded as follows: Ala in pale blue, Ile in orange, Leu in yellow, Met in red, Thr in green and Val in dark blue. Arrows indicate the path followed by AMIGO during the methyl walks.


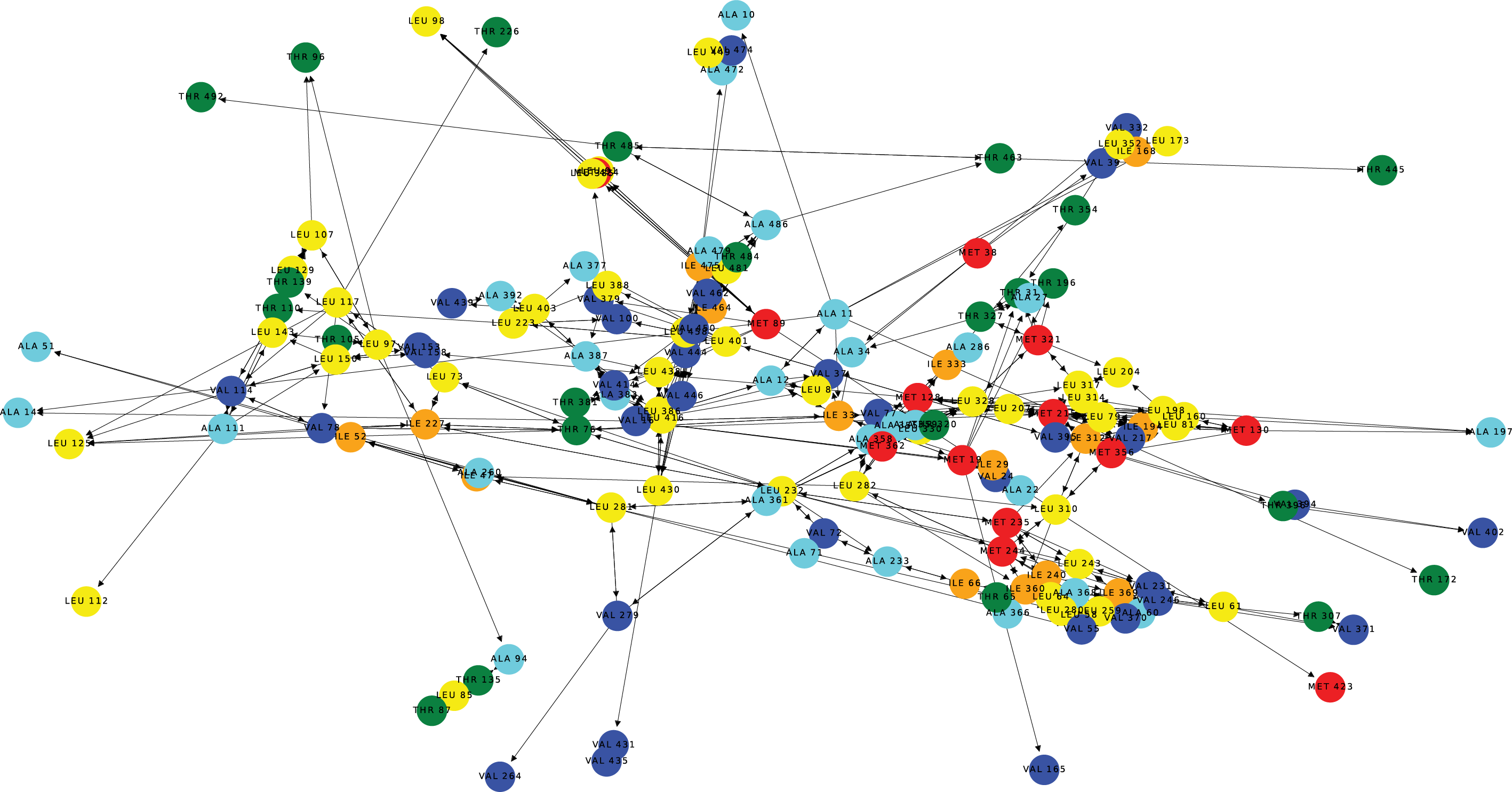


**Fig. S2** **Structure-based graph created for the protein Lm-UGP showing the methyl walks** (pdb code 4m2a). Amino acids are colour-coded as follows: Ala in pale blue, Ile in orange, Leu in yellow, Met in red, Thr in green and Val in dark blue. Arrows indicate the path followed by AMIGO during the methyl walks.

**3.3 Validation of assignment using experimentally and theoretically determined additional restraints**

For good assignment solutions, experimental and theoretical values should follow a linear correlation (in this case PCS). Such correlation figures are automatically generated by AMIGO (Figure S3). Plotting the data shows that measured and theoretically determined values are in good agreement, validating the assignment independently.

**
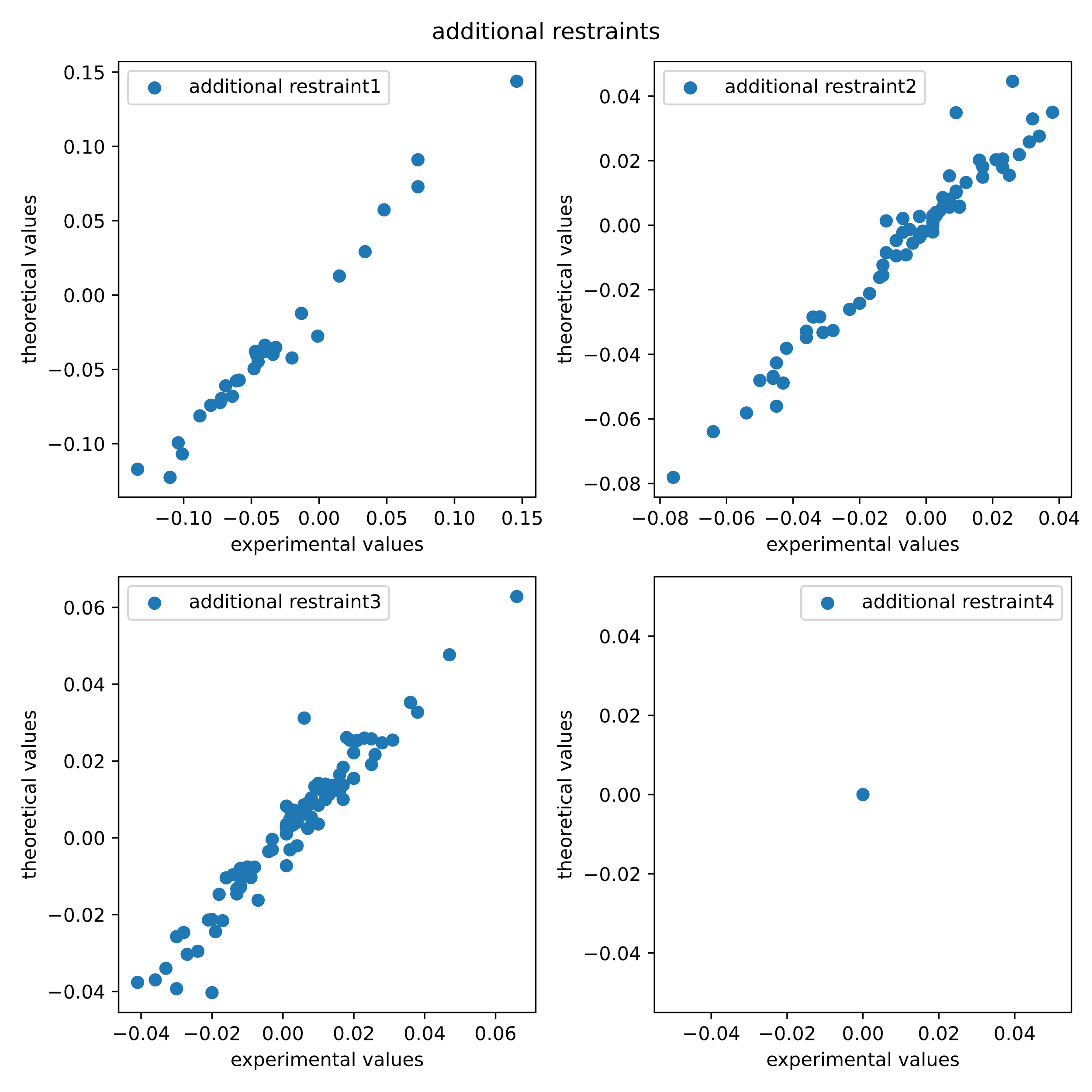
**

**Fig. S3 Example for assignment validation using experimentally and theoretically determined additional restraints (PCSs) for the LmUGP data set.** (*From top to bottom and from left to right*) Panels correspond to PCS from Tb^3+^ (additional restraint 1), Ce^3+^ (additional restraint 2) and Eu^3+^ (additional restraint 3). Values are in ppm.

**Parameter optimisation**

The following parameters were used to evaluate AMIGO’s performance across the benchmark proteins. Initially, cut-off distance ranges for each amino acid type bearing methyl groups were selected by performing test runs on each protein using only the 10% of methyl groups with the highest *rarity score* NMR graph building blocks as potential methyl walk starting points. Cut-off distances ranged from 3.0 Å to 7.0, 7.5, 8, 8.5, 9, 9.5, and 10.0 Å in 0.1 or 0.2 Å increments (Fig. S12). The NOE weighting factor $W_{NOE}$ was fixed at 1 (Eq. 5), and additional restraint weights ($W_{i}$) were set to 0 in the absence of supplementary data (Eqs. 6 and 7). For each cluster, the ratio between the assignments where the NMR graph building block exactly matched the structure graph building block and the total assignments was calculated (Fig. S12). Optimal cut-off distances—those yielding the highest exact-match ratios—were then applied in final runs using all NMR graph building blocks as possible starting points. Methyls assigned by AMIGO but lacking a reference assignment were flagged as unconfirmed.

The use of additional structural restraints such as PCS renders AMIGO less sensitive to the range of cut-off distances selected. Here, cut-off distance ranges from 3 Å to 8, 8.5, and 9 Å in 0.1 Å or 0.2 Å steps were selected. All NMR-based graph building blocks were considered as potential methyl walk starting points. The weighting factor for NOEs $W_{NOE}$ was set to 1 (Eq. 5). The weighting factors for additional restraints (different PCS data sets available for each protein) $W_{1},W_{2},W_{3}, W_{4}$ in Eq. 6 were set to 100, 100, 100 and 0 for GTB and MNV P-domain, and 0, 100, 100, 0 for LmUGP. Note that a weighting factor of 100 was used for PCS so that the contributions of NOEs and PCS to the *similarity score* are of equal magnitude. The value of 0 indicates that no data was included for that particular $W_{i}$. Methyl groups assigned by AMIGO but lacking a reference assignment in the original publication were considered unassigned.

Final runs were carried out using the same step sizes as for parameter optimisation (see above or Fig. S12) to sample the cut-off distance ranges used for the different data sets, yielding optimal results for all data sets. Larger step sizes (e.g. 0.2 or 0.5 Å) enable faster calculation times at the cost of erroneous assignments.

**Structural ensembles**

AMIGO processes each protein structure in the structural ensemble individually. For example, if structure A contains an alanine (Ala) and a methionine (Met), and structure B also contains an Ala and a Met, AMIGO generates one building block for each methyl group in each structure (Ala A and Ala B in this example). From each active node (Ala A or Ala B), edges are created to all possible neighbouring methyl groups across all structures. Thus, if both Met A and Met B lie within the cut-off distance in their respective structures, the building block will contain edges to both. Similarly, the NMR building block will contain two edges to a given neighbour if an NOE is present in both NOE datasets.

AMIGO then attempts to assign the Ala simultaneously based on both structures and both NOE datasets. If the two structures differ, the corresponding NOE patterns should also differ, and the similarity score will reflect this. In other words, structural and NMR building blocks will only match if the combined NOE data can be explained by both structures together. This approach incorporates the complete information from multiple experiments into a single run.

**How SGBBs can be combined to reconstruct any structure-based graph:**


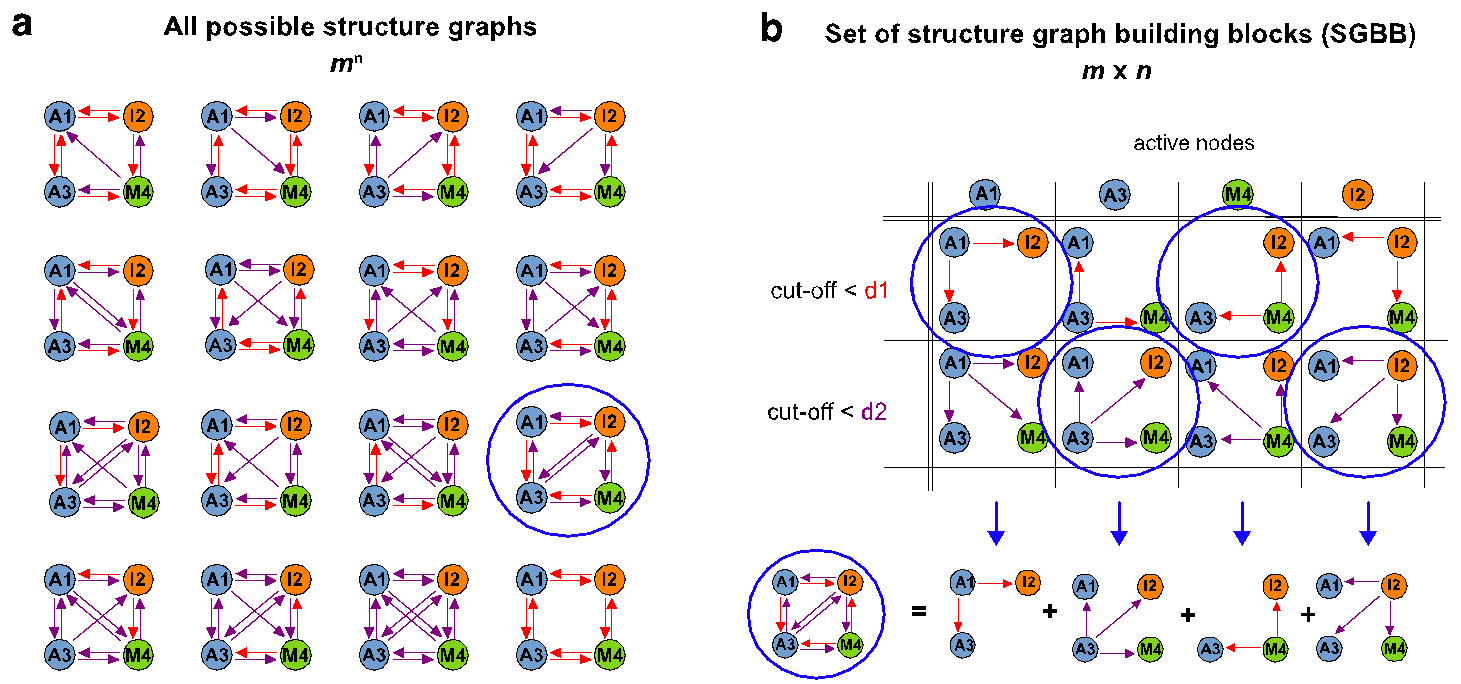


**Fig. S4** **Any possible structure graph can be created from a linear combination of structure graph building blocks (SGBB).** (a) Graph representation of all possible structure graphs for 4 nodes and 2 cutoff distances (*n^m^* = 2^4^ = 16). Nodes are labelled with a capital letter according to their amino acid type and an integer as an identifier. Deep blue large circles indicate the structure graph to be recreated using “building blocks”. (b) (*Top panel*) All possible structure graphs can be defined from eight SGBBs. Each SGBB contains one active node and all possible edges to the remaining methyl groups according to the cutoff selected. (*Bottom panel*) A linear combination of *n* = 4 building blocks (one per active node and cutoff) defines any of the 16 possible structure graphs. All nodes are colour-coded according to the amino acid as follows: orange for isoleucine (I), blue for alanine (A), and green for methionine (M).

**Constructing methyl walks from graph building blocks (GBBs) – a detailed explanation:**

Methyl walks are constructed by matching NGBBs and SGBBs following a four-step procedure. For better understanding, the method is illustrated with an example.

*Step 1: Matching an SGBB with the most similar NGBB.*

In a first step, AMIGO compares a selected NGBB with all SGBBs whose active nodes have the same amino acid type as the active node of the NGBB (Fig. S5). The comparison is evaluated by calculating the *similarity score* (Eq. 4). Briefly, the *similarity score* is based on similarities of edge patterns, adjacent amino acid types, and experimental and theoretical additional restraints, e.g., PRE or PCS. The *similarity score* is composed by the term *similarity score_NOE_*, (Eq. 5) and an additional restraints-based term or *similarity score_additional restraints_* (Eq. 6). The highest *similarity score* identifies the best matching SGBB, which will be used in Step 2 to produce the first assignment.


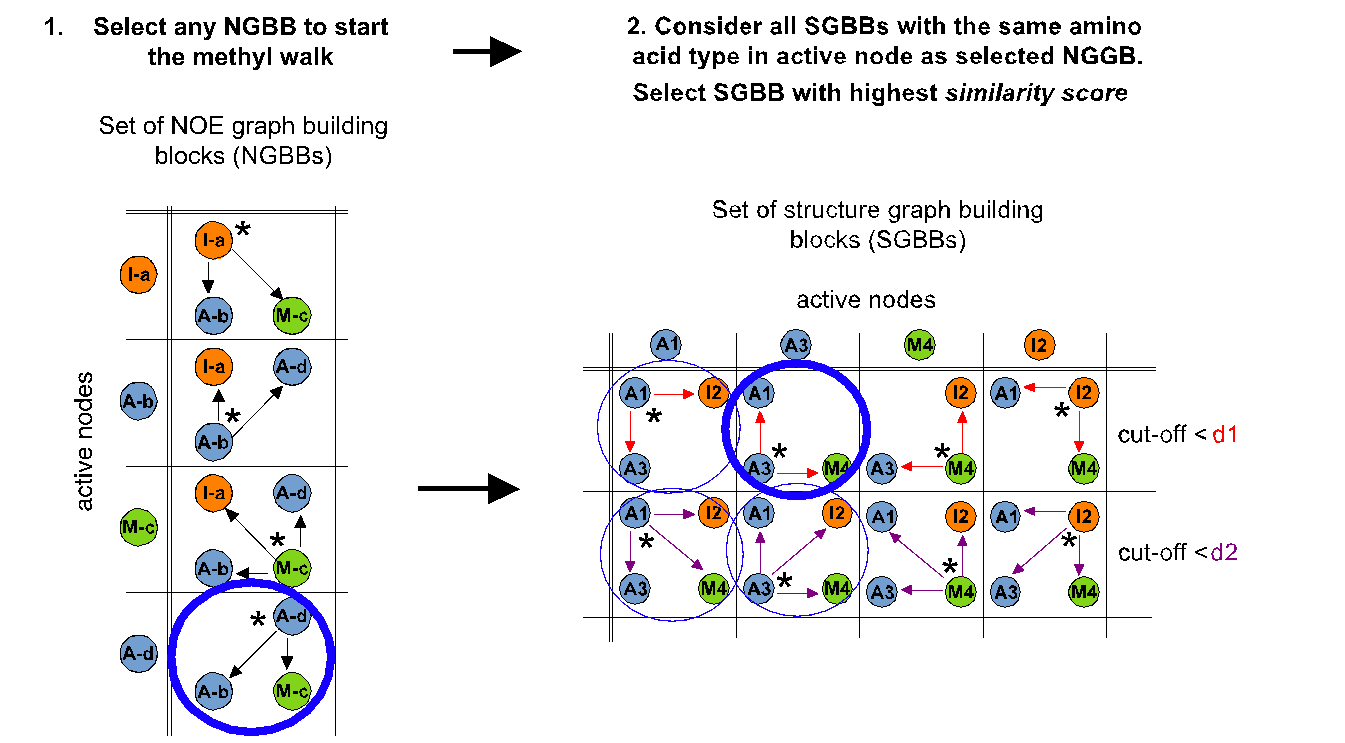


**Fig. S5 Step 1 of the identification of methyl walks from graph building blocks.** See main text to step 1 for more explanations. (*Left panel*) Decomposition of NOE graph into NGBBs. Nodes are colour-coded and labelled with a capital letter according to their amino acid type and a lower-case letter as an identifier. (*Right panel*) Identification of the closest SGGBs. Thin blue large circles indicate building blocks that are considered, while thick blue large circles highlight the building blocks that are selected. Nodes are labelled with a capital letter according to their amino acid type and an integer as an identifier. In both panels active nodes in each building block are denoted with *. All nodes are colour-coded according to the amino acid as follows: orange for isoleucine (I), blue for alanine (A), and green for methionine (M).

*Step 2: Assignment of the first resonance – the seminal assignment*

The methyl group corresponding to the active node of the SGBB with the highest *similarity score* is the first (seminal) assignment. The other nodes (cross peaks) of this NGBB suggest assignments resulting from the matching SGBB (Fig. S6). However, in this step the assignment of the nodes (cross peaks) A-b and M-c as A1 and M4 is not yet considered as set. To make assignments more likely, i.e., to set the assignments, the matching graphs need to be expanded as explained in Step 3.


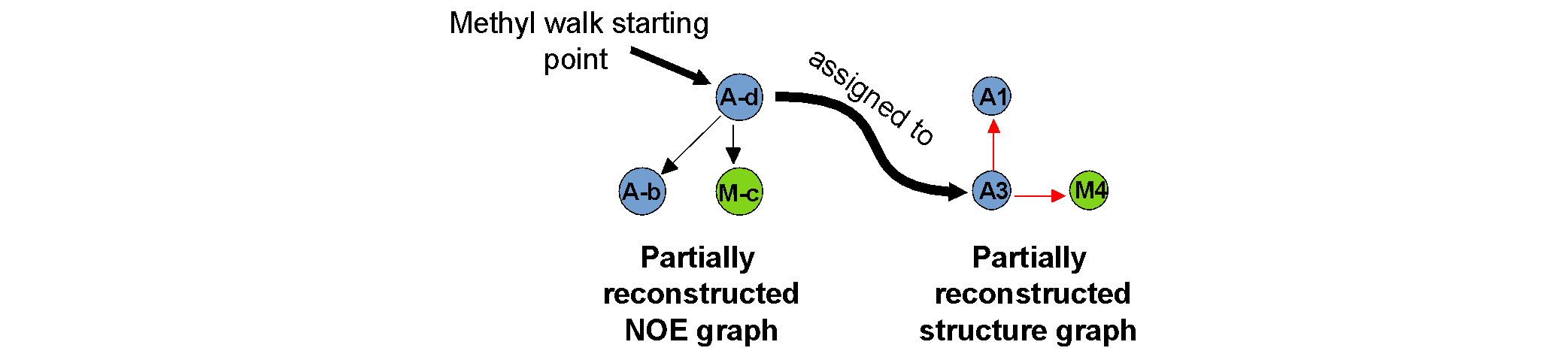


**Fig. S6 Step 2 – assignment of the first resonance.** Colour-code and node nomenclature like in Fig. S4.

*Step 3: Expanding the NOE graph using the methyl walk principle with optimal NGBB selection*

In general, Step 3 consists of identifying a node adjacent to the assigned one to be used to continue with the methyl walk. In this example (Figs. S6 and S7) the nodes (cross peaks) A-b and M-c are not assigned yet. AMIGO determines a *rarity score* (Eq. 1) for all NGBBs that have A-b or M-c as active nodes. The NGBB with the lowest *rarity* *score* of all unassigned nodes is selected for the next step next step in the methyl walk (indicated with a thick blue circle in Fig. S7). The *rarity* *score* reflects the rarity of the active node amino acid type of the NGBB within a graph. A higher score is derived from rare amino acid types and their connections to other rare amino acid types within that NGBB. Thus, the higher the *rarity score*, the more "rare" the NGBB is, which increases the probability of a correct assignment.


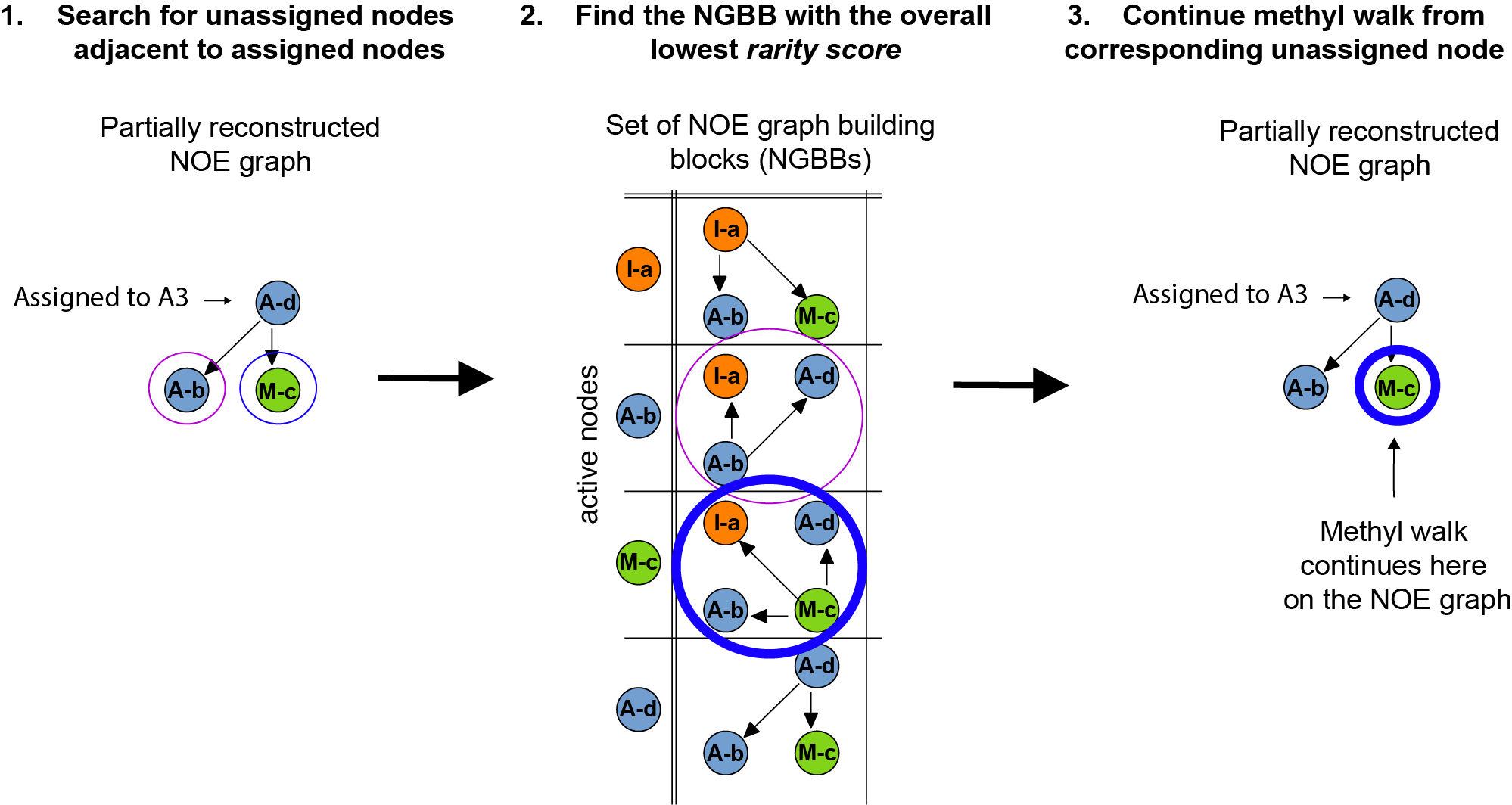


**Fig. S7 Step 3 of the identification of methyl walks from graph building blocks.** Circles with thin lines indicate the nodes (*left panel*) or NGBBs (*centre panel*) that are considered. Circles with thick lines highlight the ones being selected according to the *rarity score* (Eq. 1). Blue and violet indicate the reference unassigned nodes (A-b and M-c, respectively). Colour-code and node nomenclature like in Fig. S4.

By identifying NGBBs with active nodes that align with nodes in the partially completed NOE graph (c.f. Fig. S7; left panel), we ensure the presence of an assigned node (such as node "A-d," assigned in Step 2) within each considered NGBB. This is essential to "walk" from one assigned methyl group to the next (c.f. Fig. S7; right panel). This strategy reduces the likelihood of incorrect continuations of the methyl walk, mimicking the procedure followed by a well-trained NMR spectroscopist during manual assignments.

*Step 4: Selecting the SGBB matching the selected NGBB*

Next, the NGBB selected in Step 3 is compared to a subset of SGBBs resulting in *similarity scores* as described in Step 1. The comparison is streamlined by limiting the search to SGBBs that meet the following three criteria: i) the amino acid type of the SGBB’s active node must be identical to that of the NGBB’s active node. ii) Only SGBBs whose active nodes are unassigned and iii) correspond to a node in the partially reconstructed structure graph are considered. The corresponding nodes in the partially reconstructed structure graph naturally satisfies another criterion: they are adjacent to a node which has already been assigned to a node within the identified NGBB (for instance "A3", which is assigned to "A-d" as shown in Fig. 4). The *similarity score* (Eq. 1) is then employed to identify the best matching SGBB (Fig. S8). Importantly, this strategy significantly reduces computational time by constructing sequences of assigned nodes, equivalent to performing a classical methyl walk, within an increasingly narrow sampling space.


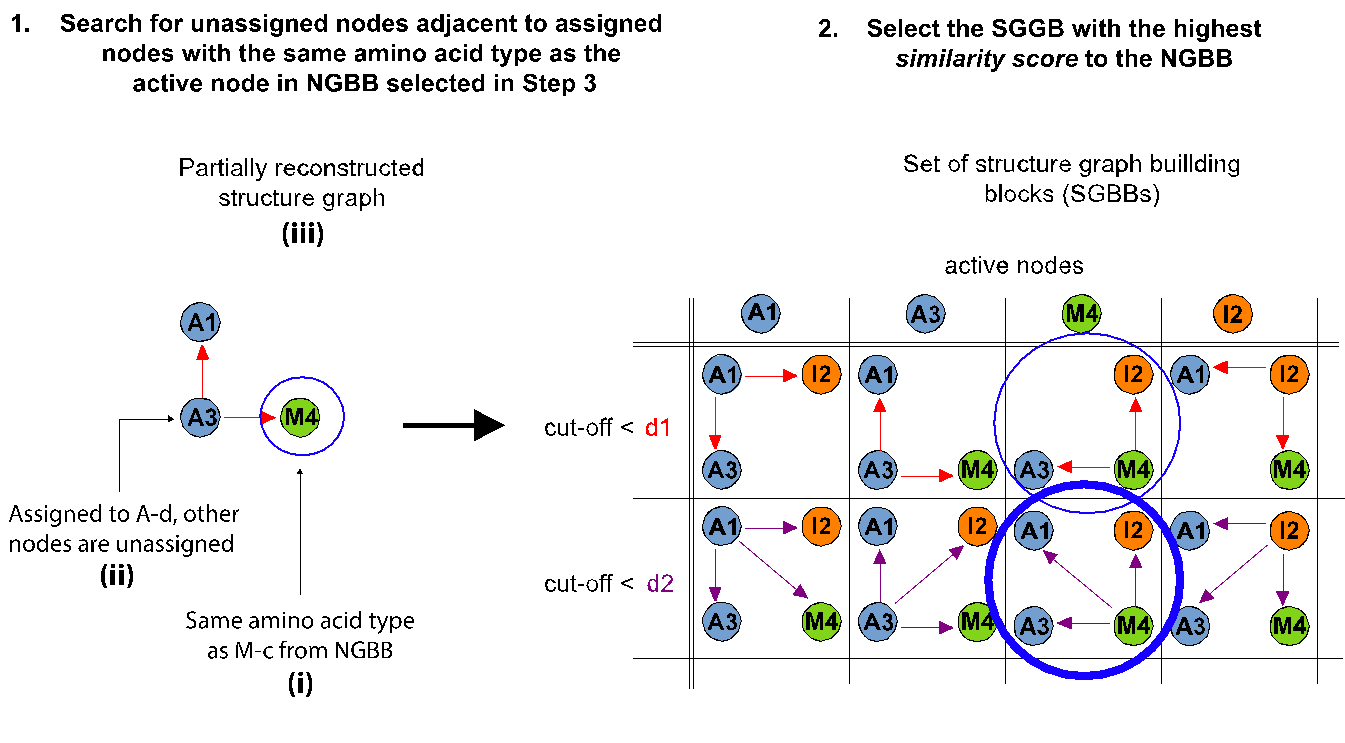


**Fig. S8 Step 4 of the identification of methyl walks from graph building blocks.** See step 4 for further explanations. Criteria i) to iii) from the text are indicated in the left panel in bold. Circles with thin lines indicate the nodes (*left panel*) or SGBBs (*right panel*) that are considered. Circles with thick lines highlight the ones being selected according to the *similarity score* (Eq. 1). Colour-code and node nomenclature like in Fig. S4.

Active nodes of the NGBB and of the best matching SGBB are assigned to each other, and the respective building blocks are used to continue the assembly of the NOE graph and a matching structure graph (Fig. S9).


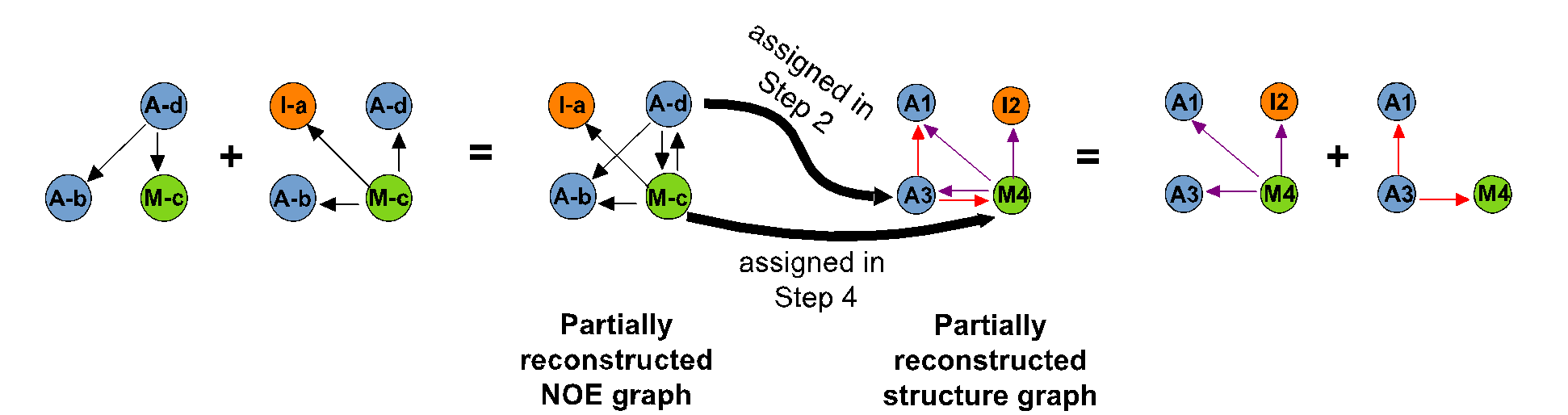


**Fig. S9 The methyl walk advances by iteration from Step 2.** Colour-code and node nomenclature like in Fig. S4.

AMIGO iterates through Steps 2, 3, and 4 until no more assignments are possible. Each methyl group is evaluated as a potential starting point for a methyl walk, leading to the generation of distinct methyl walks for each NGBB. The walks and their corresponding assignments are then assessed by the *total score* (Equation 8). The methyl walk yielding the highest *total score* is considered as the final assignment. Typically, a single methyl walk encompasses only a portion of the methyl groups present in the protein due to either connectivity gaps or unfavourable protein dynamics. To overcome this limitation, the algorithm excludes the assigned methyl groups and their corresponding NMR resonances from subsequent analysis, and the process is repeated with the remaining methyl groups until no further assignments can be performed (Fig. 2).

**Examples illustrating the calculation of *similarity* and *rarity* scores:**


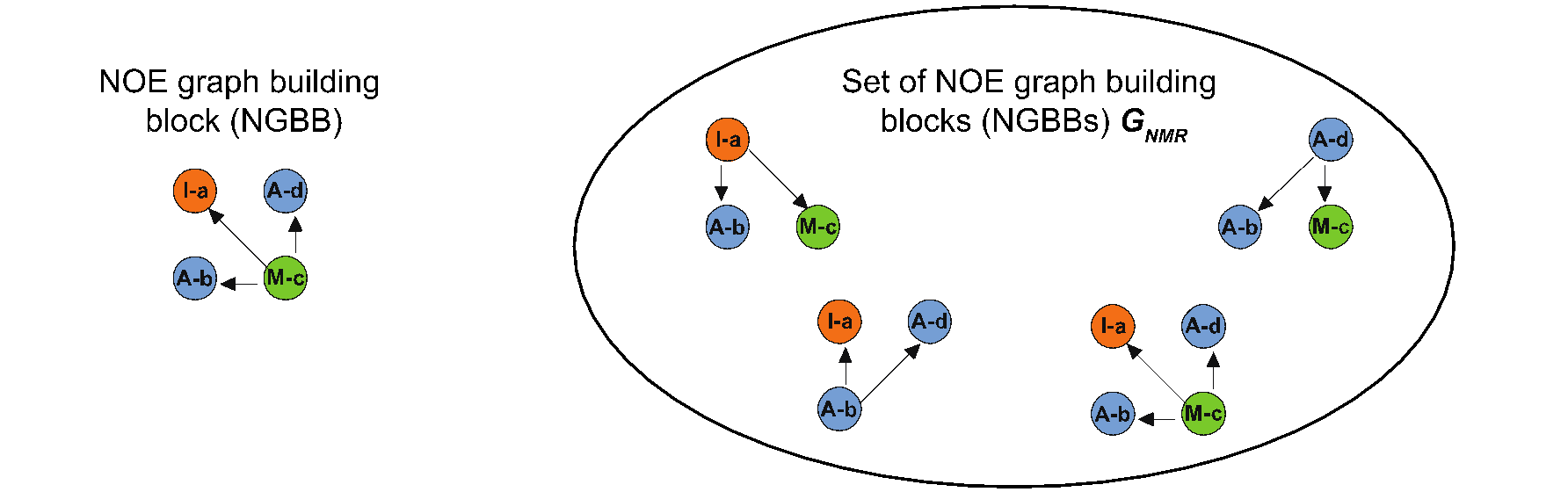


**Fig. S10 Example of calculation of the *rarity score*.** The *rarity score* is calculated for a particular NOE graph building block (*left panel*, NGBB) being part of a set of NGBBs termed $G_{NMR}$ (*right panel*) as described in Eq. 1. Here, *k* = 3, $n_{NGBB}$ = 1, and $n_{total}$ = 4. According to Eq. 1, $p_{active}$ = ¼. The first, second and third neighbour of the active node in the selected NGBB (*left panel*) are A-d, I-a and A-b and have the amino acid type alanine, isoleucine and alanine, respectively. $p_{neighbor,1}$ corresponding to the first neighbor, i.e., A-d, is then calculated according to Eq. 3 as follows: as there are 5 edges from an active node to an alanine ($n_{neighbor,1}$) and 9 edges in total ($n_{total,neighbor}$) in the set of NGBBs $G_{NMR}$, $p_{neighbor,1}$ = 5/9. $p_{neighbor,2}$ and $p_{neighbor,3}$ are calculated in the same way. This results in a *rarity score* of -2.7x10^-4^ for the particular NGBB in the *left panel*, making this building block the one with the highest *rarity score* in $G_{NMR}$.


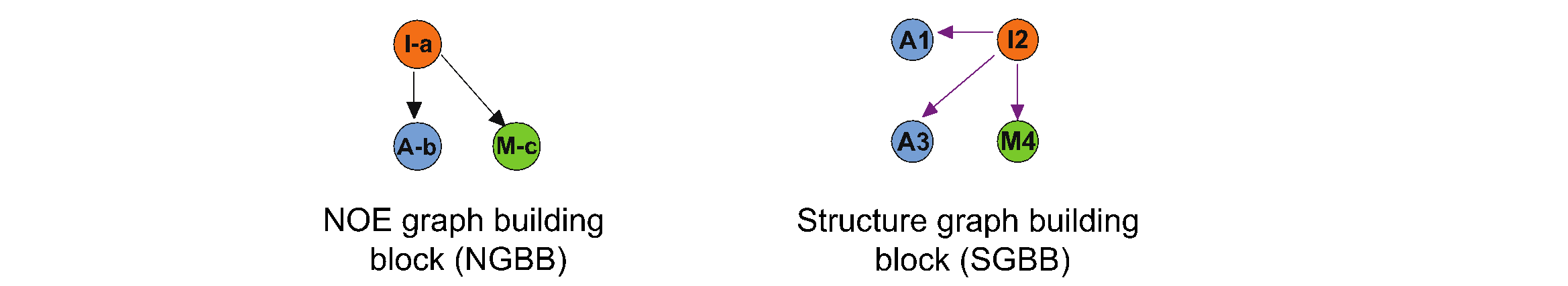


**Fig. S11 Exemplary calculation of the *similarity score_NOE_*.** This simple example compares a NOE graph building block (*left panel*, NGBB) with a structure graph building block (*right panel*, SGGB) to calculate their degree of similarity as a function of their *similarity score_NOE_*. Nodes in orange, blue and green and labels I, A and M indicate the amino acid type as isoleucine, alanine and methionine, respectively. Letters a to c in NGBB and numbers 1 to 4 in SGBB uniquely identify nodes in their corresponding building block. According to Eq. 5, the number of common edges *c* is 2, as there is an edge from an isoleucine node to a methionine node and an edge from an isoleucine node to an alanine node occurring in both building blocks. The number of total edges $E_{NOE}$ and $E_{structure}$ are 2 and 3, respectively. As a result, the *similarity score_NOE_* of the pair of building blocks = ${2(2W}_{NOE})-\left| 2-2 \right|-\left| 2-3 \right|=3$, with $W_{NOE}=1.$

#### **About the assignment of methyl groups in close proximity to paramagnetic metals:**

#### **Hidden safeguard 1: lower bound on the similarity score (default = -100)**

*What it does:*

Each time AMIGO compares an NGBB with an SGBB it computes a *similarity score* (Eq. 1).
If PCS restraints are included and the measured PCS (PCS_exp_) deviates strongly from the PCS predicted from the current tensor (PCS_calc_), a penalty term is added (Eq. S1):

$$penalty=\omega_{PCS}\left| {PCS}_{exp}-{PCS}_{calc} \right|$$

where $\omega_{PCS}$ is defined as –100. For methyl groups within ≲ 10 Å of the paramagnetic ion the PCS gradient is steep and even a 0.5 ppm mismatch can push the overall similarity score below –100. To keep the search space tractable the code rejects any candidate with a score below a hard floor (-100 by default). The assumption was that “real” matches would always score higher.

*Why this matters:*

If many near-centre methyls are present, or $\omega_{PCS}$ is set higher than -100, true assignments can be discarded prematurely. We have tested hard floors as low as –100000 in the GTB test to verify this behaviour; the change alone did not restore the missing assignments around the catalytic active site, indicating that large deviation between PCS_exp_ and PCS_calc_ were present around the paramagnetic metal.

**Hidden safeguard 2: lower bound on the *total score* (default = 0)**

*What it does:*

Once a methyl walk is completed, AMIGO sums individual similarity scores to obtain a *total* score (Eq. 8). Walks starting from a single edge, or containing many PCS-dominated penalties, can end up negative. Any walk whose total score ≤ 0 is discarded by the program.

*Why this matters:*

For GTB the methyl groups located in the carbohydrate binding pocket formed very short walks and suffered large PCS mismatches (see above), giving negative *total scores* and triggering the filter. Consequently, no assignment was performed unless additional information (pre-assignments) was supplied or PCS weighting was switched off.

**Troubleshooting:**
In general, PCS from sites closer than ≲ 10 Å to the metal should be downweighted or omitted unless the magnetic susceptibility tensor is extremely well characterised. Large PCS errors at such positions can arise from uncertainty in tensor orientation/magnitude (especially with sparse PCS data), local backbone mobility or alternate rotamers, residual electron-spin relaxation effects. Removing or downweighting these PCS terms reduce false penalties and improves robustness across all automated assignment tools, not just AMIGO. Therefore, after completing an AMIGO run with PCS we selected all performed assignments as pre-assignments and re-run AMIGO without PCS. This resulted in 12 out of 16 methyls located in the carbohydrate binding site being assigned. As a rule, when methyl groups close to paramagnetic metals become consistently unassigned, we recommend the following:

1) Pre-assign all non-loop methyls
This anchors the walks and prevents incorrect permutations that would otherwise mask the loop residues.

2) Disable PCS input for the final run
With the big PCS penalties removed, *similarity* and *total scores* remain above both thresholds, allowing AMIGO to propagate into the loop regions.

**How we finally recovered the loop assignments in GTB**

1. **Pre-assign all non-loop methyls.**
   This anchors the walks and prevents incorrect permutations that would otherwise mask the loop residues.
2. **Disable PCS input for the final pass.**
   With the big PCS penalties removed, similarity and total scores remain above both thresholds, allowing AMIGO to propagate into the loop regions.
3. **Keep safeguard 2 active.**
   This ensures any residual noise-driven walks with net negative support are still filtered out.

**
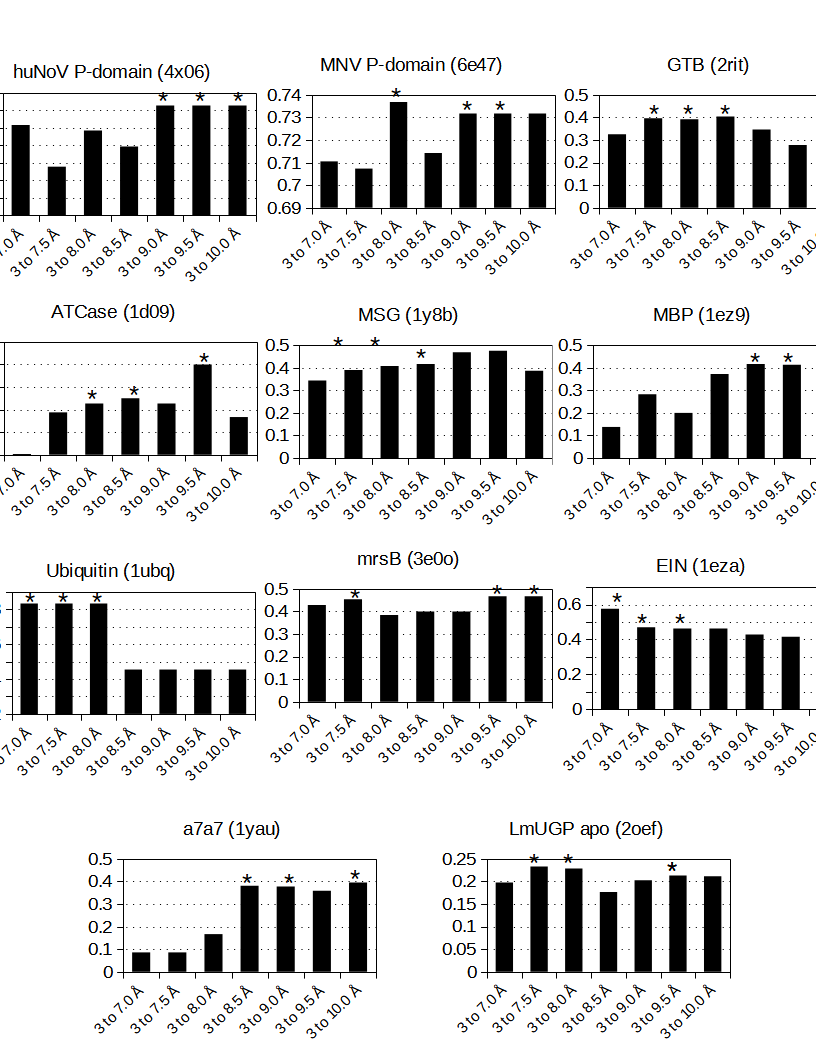
**

**Fig. S12 Test runs for determination of optimal range of cut-off distances for the benchmark set in the absence of additional restraints.** The step size from the minimal to maximal cut-off distance was 0.1 Å except for a7a7, GTB, and LmUGP apo, where a step size of 0.2 Å was used. The range of cut-off distances was optimised for each protein. Selected cut-off ranges are indicated with an *, and correspond to the run with the highest *N_match_*/*N_total_* ratio. *N_total_* corresponds to the total number of assigned resonances, while *N_match_* denotes resonances for which the NOE building block and the corresponding structure building block matched perfectly. Selected cut-off ranges were used to run the final assignments.

**Table S1: Reference assignments for the tested experimental data sets.**

| **Entry N°** | **Protein** | **References for data and assignment** | **PDB code of structural model** |
| --- | --- | --- | --- |
| 1 | mrsB | (Lange et al. 2012; Pritišanac et al. 2017) | 3e0o (Kim et al. 2009) |
| 2 | Ubiquitin | (Chao et al. 2012; Pritišanac et al. 2017) | 1ubq (Vijay-kumar et al. 1987) |
| 3 | EIN | (Venditti et al. 2011; Pritišanac et al. 2017) | 1eza (Garrett et al. 1997) |
| 4 | MBP | (Lange et al. 2012; Pritišanac et al. 2017) | 1ez9 (Duan and Quiocho 2002) |
| 5 | msg | (Tugarinov and Kay 2003; Tugarinov et al. 2005; Pritišanac et al. 2017) | 1y8b (Tugarinov et al. 2005) |
| 6 | ATCase | (Velyvis et al. 2009; Pritišanac et al. 2017) | 1d09 (Jin et al. 1999) |
| 7 | a7a7 | (Tugarinov et al. 2007; Pritišanac et al. 2017) | 1yau (Förster et al. 2005) |
| 8 | HuNoV P-domain | (Müller-Hermes et al. 2020) | 4x06 (Singh et al. 2015)* |
| 9 | MNV P-domain | (Maass et al. 2022) | 6e47 (Nelson et al. 2018) |
| 10 | LmUGP | (Mühlberg et al. 2022) | 4m2a (Führing et al. 2013)** |
| 11 | GTB | (Flügge and Peters 2018, 2019) | 2rit (Alfaro et al. 2008)** |

*Crystal structure was minimized using Maestro as described in the corresponding manuscript.

**Crystal structures were modified as described in the corresponding manuscript.

**Table S2: Proteins used to create PDB-based synthetic NOE networks.**

| **Entry N°** | **Protein** | **Molecular weight (kDa)** | **PDB code of structural model** |
| --- | --- | --- | --- |
| 1 | Trypsin | 23.7 | 8izk |
| 2 | Tetanus toxin c | 52.2 | 1a8d |
| 3 | Phosphorylase | 91.4 | 1v7v |
| 4 | TYRP1 | 54.2 | 9ey7 |
| 5 | TEAD1 chain A | 52.1 | 7li5 |
| 6 | Glu cyclase apo chain A | 117.6 | 9fxg |
| 7 | Fatty acid elongase chain A | 73.0 | 6y7f |
| 8 | Carbonic anhydrase XIII | 60.0 | 3d0n |

**Bibliography:**

Alfaro JA, Zheng RB, Persson M, et al (2008) ABO(H) Blood Group A and B Glycosyltransferases Recognize Substrate via Specific Conformational Changes. Journal of Biological Chemistry 283:10097–10108. https://doi.org/10.1074/jbc.M708669200

Chao F-A, Shi L, Masterson LR, Veglia G (2012) FLAMEnGO: A fuzzy logic approach for methyl group assignment using NOESY and paramagnetic relaxation enhancement data. Journal of Magnetic Resonance 214:103–110. https://doi.org/10.1016/j.jmr.2011.10.008

Duan X, Quiocho FA (2002) Structural Evidence for a Dominant Role of Nonpolar Interactions in the Binding of a Transport/Chemosensory Receptor to Its Highly Polar Ligands ^,^. Biochemistry 41:706–712. https://doi.org/10.1021/bi015784n

Flügge F, Peters T (2018) Complete assignment of Ala, Ile, Leu, Met and Val methyl groups of human blood group A and B glycosyltransferases using lanthanide-induced pseudocontact shifts and methyl–methyl NOESY. J Biomol NMR 70:245–259. https://doi.org/10.1007/s10858-018-0183-4

Flügge F, Peters T (2019) Insights into Allosteric Control of Human Blood Group A and B Glycosyltransferases from Dynamic NMR. ChemistryOpen 8:760–769. https://doi.org/10.1002/open.201900116

Förster A, Masters EI, Whitby FG, et al (2005) The 1.9 Å Structure of a Proteasome-11S Activator Complex and Implications for Proteasome-PAN/PA700 Interactions. Molecular Cell 18:589–599. https://doi.org/10.1016/j.molcel.2005.04.016

Führing J, Cramer JT, Routier FH, et al (2013) Catalytic Mechanism and Allosteric Regulation of UDP-Glucose Pyrophosphorylase from Leishmania major. ACS Catal 3:2976–2985. https://doi.org/10.1021/cs4007777

Garrett DS, Seok Y-J, Liao D-I, et al (1997) Solution Structure of the 30 kDa N-Terminal Domain of Enzyme I of the *Escherichia coli* Phosphoenolpyruvate:Sugar Phosphotransferase System by Multidimensional NMR. Biochemistry 36:2517–2530. https://doi.org/10.1021/bi962924y

Jin L, Stec B, Lipscomb WN, Kantrowitz ER (1999) Insights into the mechanisms of catalysis and heterotropic regulation ofEscherichia coli aspartate transcarbamoylase based upon a structure of the enzyme complexed with the bisubstrate analogueN-phosphonacetyl-L-aspartate at 2.1 ? Proteins 37:729–742. https://doi.org/10.1002/(SICI)1097-0134(19991201)37:4<729::AID-PROT21>3.0.CO;2-F

Kim YK, Shin YJ, Lee W, et al (2009) Structural and kinetic analysis of an MsrA–MsrB fusion protein from *Streptococcus pneumoniae*. Molecular Microbiology 72:699–709. https://doi.org/10.1111/j.1365-2958.2009.06680.x

Lange OF, Rossi P, Sgourakis NG, et al (2012) Determination of solution structures of proteins up to 40 kDa using CS-Rosetta with sparse NMR data from deuterated samples. Proc Natl Acad Sci USA 109:10873–10878. https://doi.org/10.1073/pnas.1203013109

Maass T, Westermann LT, Creutznacher R, et al (2022) Assignment of Ala, Ile, LeuproS, Met, and ValproS methyl groups of the protruding domain of murine norovirus capsid protein VP1 using methyl–methyl NOEs, site directed mutagenesis, and pseudocontact shifts. Biomol NMR Assign 16:97–107. https://doi.org/10.1007/s12104-022-10066-7

Mühlberg L, Alarcin T, Maass T, et al (2022) Ligand-induced structural transitions combined with paramagnetic ions facilitate unambiguous NMR assignments of methyl groups in large proteins. J Biomol NMR 76:59–74. https://doi.org/10.1007/s10858-022-00394-0

Müller-Hermes C, Creutznacher R, Mallagaray A (2020) Complete assignment of Ala, Ile, LeuProS, Met and ValProS methyl groups of the protruding domain from human norovirus GII.4 Saga. Biomol NMR Assign 14:123–130. https://doi.org/10.1007/s12104-020-09932-z

Nelson CA, Wilen CB, Dai Y-N, et al (2018) Structural basis for murine norovirus engagement of bile acids and the CD300lf receptor. Proc Natl Acad Sci USA 115:. https://doi.org/10.1073/pnas.1805797115

Pritišanac I, Degiacomi MT, Alderson TR, et al (2017) Automatic Assignment of Methyl-NMR Spectra of Supramolecular Machines Using Graph Theory. J Am Chem Soc 139:9523–9533. https://doi.org/10.1021/jacs.6b11358

Singh BK, Leuthold MM, Hansman GS (2015) Human Noroviruses’ Fondness for Histo-Blood Group Antigens. J Virol 89:2024–2040. https://doi.org/10.1128/JVI.02968-14

Tugarinov V, Choy W-Y, Orekhov VYu, Kay LE (2005) Solution NMR-derived global fold of a monomeric 82-kDa enzyme. Proc Natl Acad Sci USA 102:622–627. https://doi.org/10.1073/pnas.0407792102

Tugarinov V, Kay LE (2003) Ile, Leu, and Val Methyl Assignments of the 723-Residue Malate Synthase G Using a New Labeling Strategy and Novel NMR Methods. J Am Chem Soc 125:13868–13878. https://doi.org/10.1021/ja030345s

Tugarinov V, Sprangers R, Kay LE (2007) Probing Side-Chain Dynamics in the Proteasome by Relaxation Violated Coherence Transfer NMR Spectroscopy. J Am Chem Soc 129:1743–1750. https://doi.org/10.1021/ja067827z

Velyvis A, Schachman HK, Kay LE (2009) Assignment of Ile, Leu, and Val Methyl Correlations in Supra-Molecular Systems: An Application to Aspartate Transcarbamoylase. J Am Chem Soc 131:16534–16543. https://doi.org/10.1021/ja906978r

Venditti V, Fawzi NL, Clore GM (2011) Automated sequence- and stereo-specific assignment of methyl-labeled proteins by paramagnetic relaxation and methyl–methyl nuclear overhauser enhancement spectroscopy. J Biomol NMR 51:319–328. https://doi.org/10.1007/s10858-011-9559-4

Vijay-kumar S, Bugg CE, Cook WJ (1987) Structure of ubiquitin refined at 1.8 Å resolution. Journal of Molecular Biology 194:531–544. https://doi.org/10.1016/0022-2836(87)90679-6
